# Supplementary material for: MeCP2 regulates gene expression through recognition of H3K27me3
Source: Nat Commun. 2020 Jun 19;11:3140. doi: 10.1038/s41467-020-16907-0 (PMC7305159; doi:10.1038/s41467-020-16907-0)
Supplement: Supplementary file 1 — Supplementary Information [file 41467_2020_16907_MOESM1_ESM.pdf]

# MeCP2 regulates gene expression through recognition of H3K27me3

Lee *et al.*

**Supplementary Material:**

Supplementary Figures 1-10

Supplementary Tables 1-7

Supplementary Methods

Supplementary References

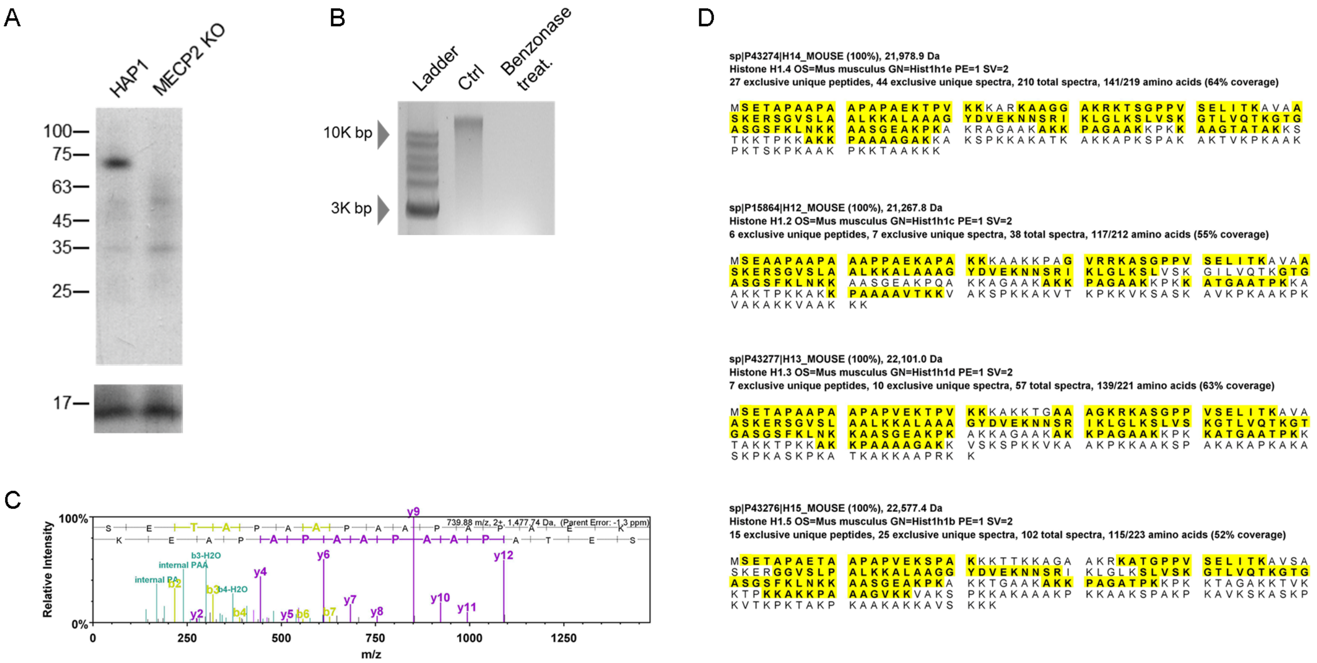


**Supplementary Fig. 1. Identification of MeCP2 binding proteins.** (A) Rabbit MeCP2 antibody (Diagenode, pAb-052-050) specificity was tested by its ability to recognize a band at about 75 kD. Nucleus extracts from HAP1 cells and *MECP2* knockout HAP1 cell line (Horizon, HZGHC001102c010) were analyzed by Western blot using the MeCP2 antibody. Loading quantities were monitored by blotting histone H3 in each lane (lower panels). Source data are provided as a Source Data file. (B) Completeness of Benzonase treatment is shown on 1% agarose gel with untreated nuclear extracts as control. (C) MALDI-TOF mass spectrometry analysis of the 25-30 kDa band shown in Fig. 1A identifies histone H1. (D) Peptides of the sample overlapping with histone H1 isoforms are highlighted in yellow.

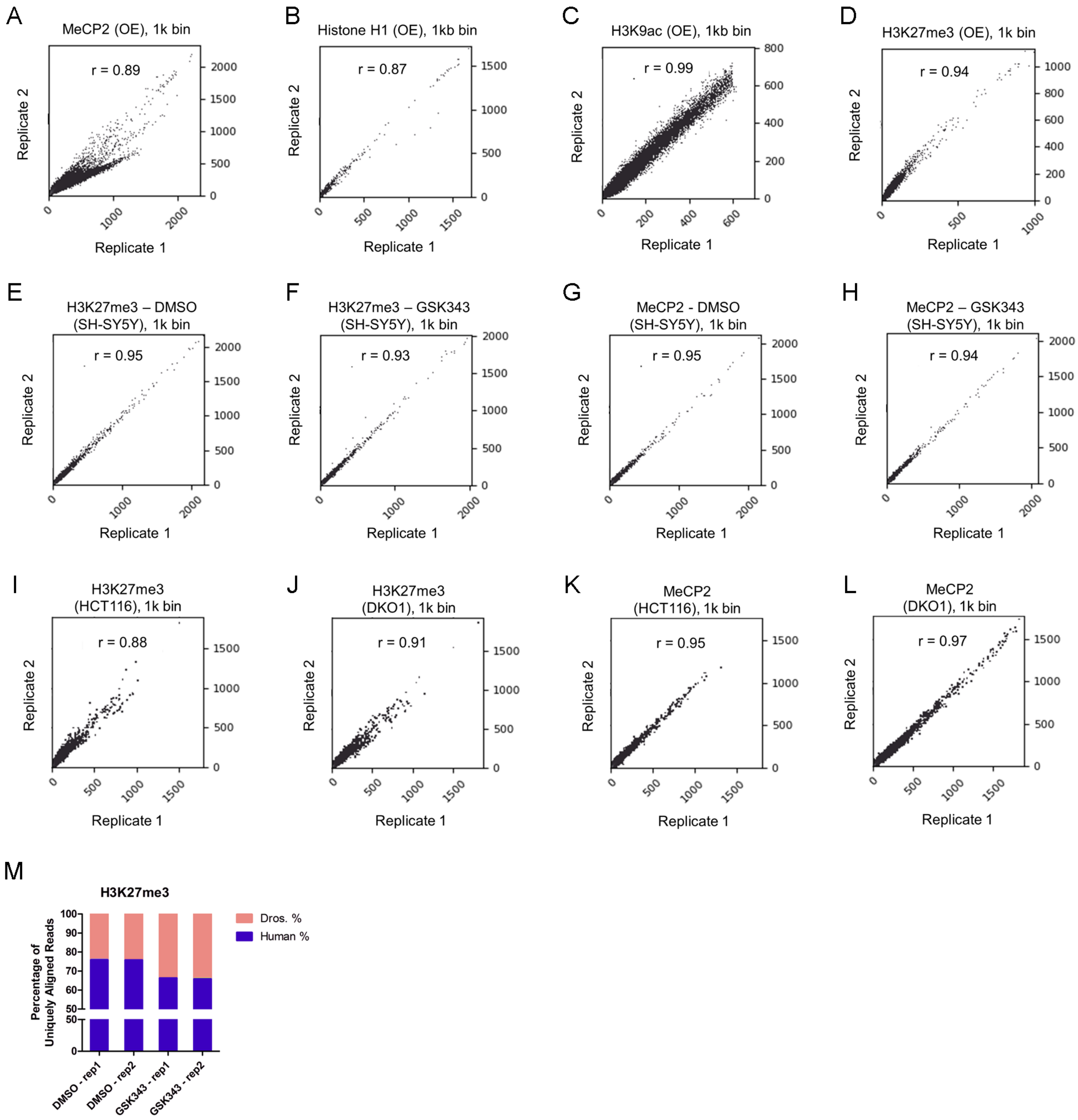


**Supplementary Fig. 2. Reproducibility of ChIP-seq signals between two biological replicates.** (A-L) Scatter plots of ChIP-seq genome coverage show a high correlation between the replicates (1000-bp windows). (A) MeCP2 ChIP-seq from OE tissue, (B) histone H1 ChIP-seq from OE tissue, (C) H3K9ac ChIP-seq from OE tissue, (D) H3K27me3 ChIP-seq from OE tissue, (E) H3K27me3 ChIP-Rx from DMSO-treated SH-SY5Y cells, (F) H3K27me3 ChIP-Rx from GSK343-treated SH-SY5Y cells, (G) MeCP2 ChIP-Rx from DMSO-treated SH-SY5Y cells, and (H) MeCP2 ChIP-Rx from GSK343-treated SH-SY5Y cells. (I) H3K27me3 ChIP-Rx from HCT116 cells, (J) H3K27me3 ChIP-Rx from DKO1 cells, (K) MeCP2 ChIP-Rx from HCT116 cells, and (L) MeCP2 ChIP-Rx from DKO1 cells. The biological duplicates of ChIP-seq data demonstrated a good agreement between the samples; therefore, the duplicates were combined for downstream analysis. (M) Percentage of ChIP-Rx from SH-SY5Y cells treated with DMSO or GSK343 aligned with H3K27me3 human (human) or Drosophila genome (reference).

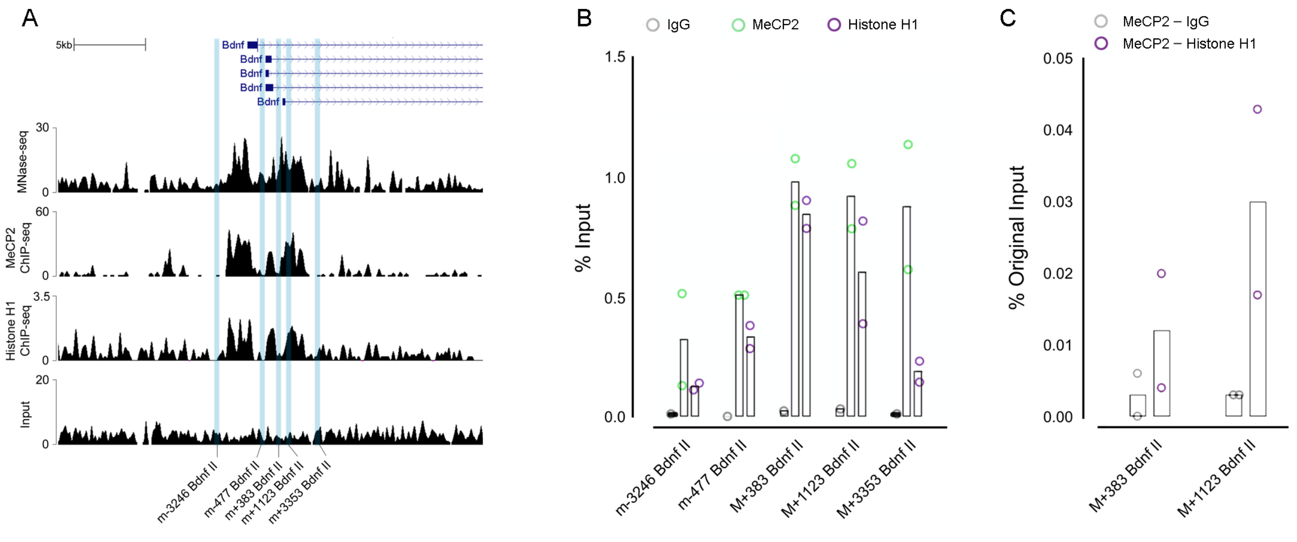


**Supplementary Fig. 3. Co-occupancy of MeCP2 and histone H1.** (A) ChIP-seq genome browser view (mm9) of MeCP2-, histone H1 occupancy, and nucleosome at *Bdnf* flanking regions. ChIP-seq signal intensity is indicated on the y-axis. Sky blue bars indicate loci for ChIP-qPCR validation. (B) ChIP-qPCR of selected regions. N = 2 (WT) biologically independent experiments. Percentage of Input are shown for no antibody control, MeCP2 ChIP and histone H1 ChIP. (C) Histone H1 ChIP on MeCP2 ChIPed protein-DNA complex are quantified by qPCR and compared to control. N = 2 (WT) biologically independent experiments. Graphs with individual data show the mean in two pairs of independent experiments (B and C).

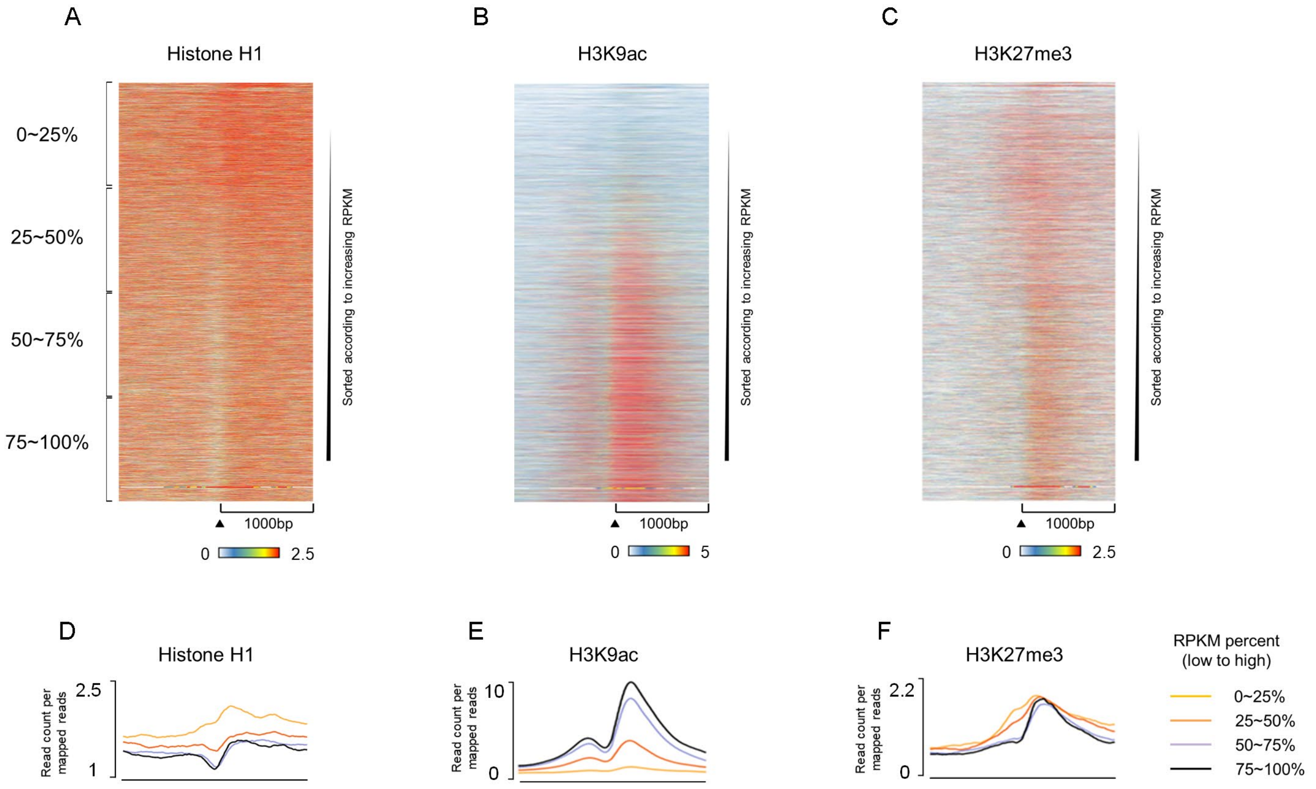


**Supplementary Fig. 4. Genomic characterization of histone H1, H3K9ac, and H3K27me3 around TSS.** (A-C) Heat maps of histone H1 (A), H3K9ac (B), and H3K27me3 (C) ChIP-seq signal intensity around TSS (-1000 to +1000 bp). Genes are ordered from lowest to highest expression, top to bottom. (D-F) The average ChIP-seq profile of histones H1 (D), H3K9ac (E), and H3K27me3 (F) around TSS in a quantile classified by gene expression. 0~25% (yellow), 25~50% (orange), 50~75% (purple), and 75~100% (black). These histone modification patterns are consistent with published studies[1-3].

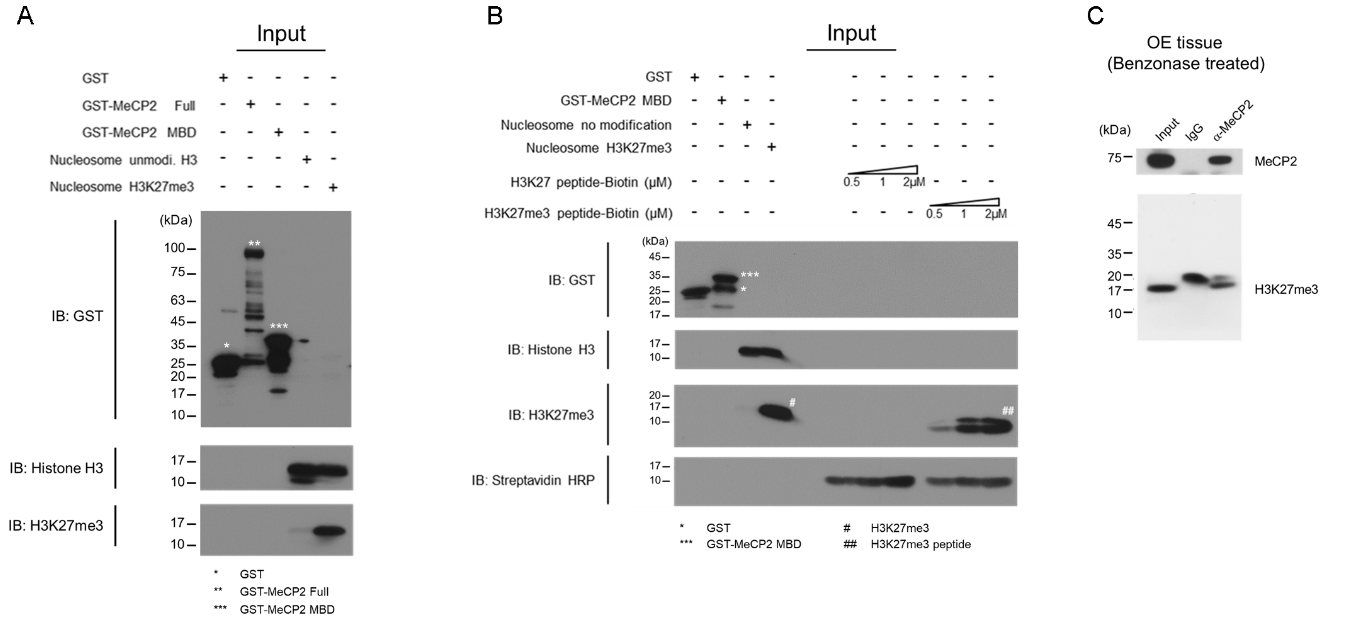


**Supplementary Fig. 5. Analyses of MeCP2 binding to H3K27me3 *in vitro* and *in vivo*.** (A and B) The presence of bait and prey proteins are shown by immunoblotting with anti-GST, anti-Histone H3, anti-H3K27me3, or Streptavidin HRP. Star, GST; Double star, GST-MeCP2 Full; Triple star, GST-MeCP2 MBD; Sharp, H3K27me3; Double sharp, H3K27me3 peptide. (C) H3K27me3 coimmunoprecipitates with MeCP2 in olfactory epithelium nuclear extracts.

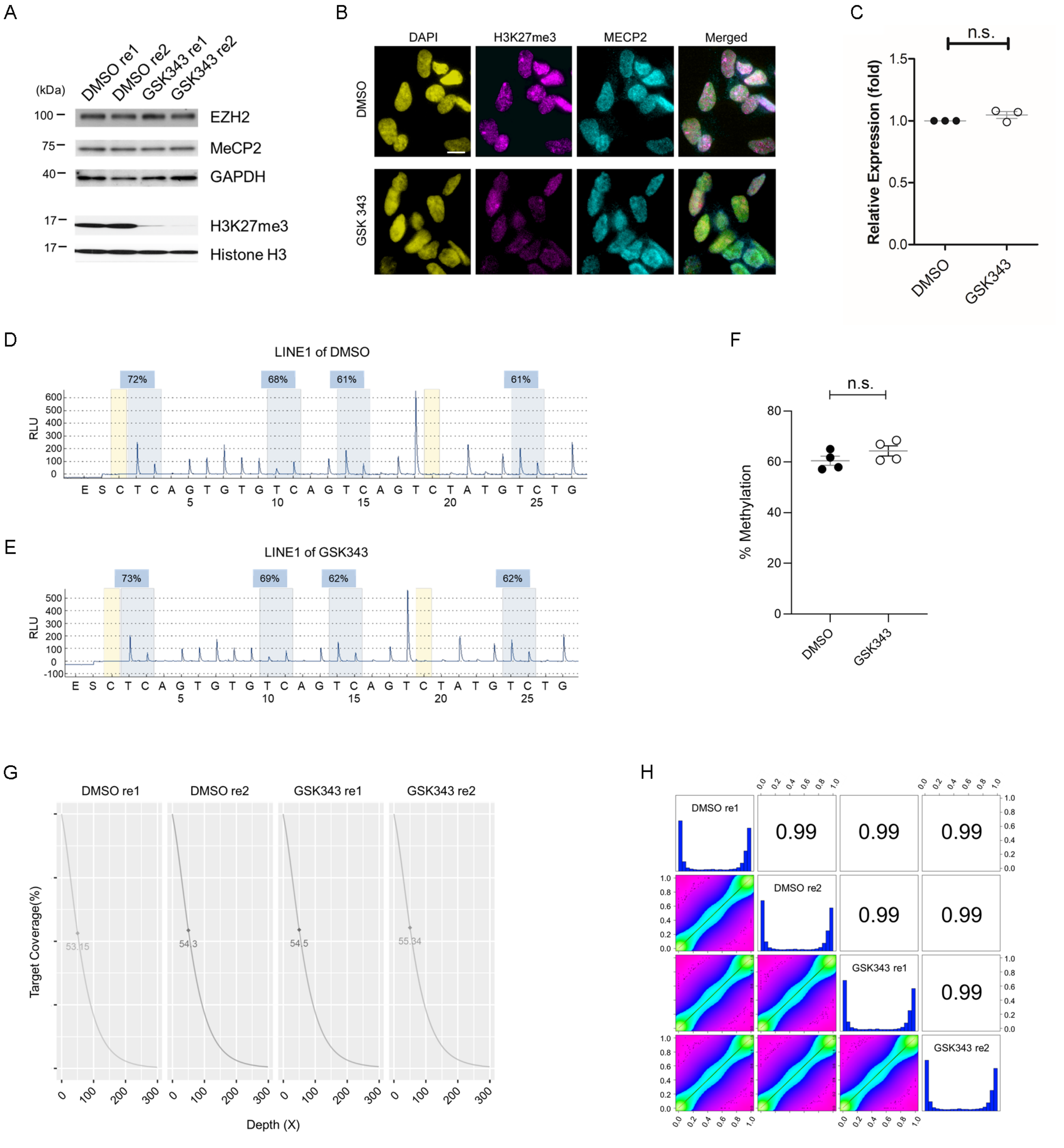


**Supplementary Fig. 6. GSK343 effect on DNA methylation.** (A) Western blot showing a global decrease in H3K27me3 in SH-SY5Y cells treated with GSK343 for 72 hr. Anti-Histone H3, and GAPDH used as loading controls. Source data are provided as a Source Data file. (B) H3K27me3 and MeCP2 immunoreactivity of either DMSO- or GSK343-treated SH-SY5Y cells. Bar = 15 μm. (C) *Mecp2* transcript levels were plotted demonstrating no significant changes in GSK343 treated cells after 72 hours, with the relative expression level of *Mecp2* in DMSO cells defined as 1. N = 3 (per treatment) biologically independent experiments. $p = 0.177$, $t_4 = 1.639$ (Two-tailed t-test). Graph

with individual data show average ± s.e.m. (D-F) Bisulfite Pyrosequencing analysis for LINE-1 methylation of (D) DMSO- or (E) GSK343-treated SH-SY5Y cells. The x axis corresponds to Pyrosequencing of LINE1 loci after bisulfite conversion; the y axis corresponds to relative light unit (RLU). The "C %" numbers (in dark shade) are proportions of C and T, representing methylated cytosine (5mC) and unmethylated cytosine (C), at each CpG site after bisulfite conversion. The yellow shades indicate bisulfite conversion control for complete bisulfite conversion. (F) Bisulfite Pyrosequencing analysis for LINE-1 methylation of DMSO- or GSK343-treated SH-SY5Y cells. N = 4 (per treatment, DMSO vs GSK343) from biologically independent experiments, $p = 0.20$, $t_6 = 1.432$ (Two-tailed t-test). Graph with individual data show average ± s.e.m. (G and H) Targeted bisulfite sequencing of DMSO or GSK343 treated SH-SY5Y cells. N = 2 (per treatment, DMSO vs GSK343) from biologically independent experiments. (G) Coverage plot showing target coverage rate by mean depth. The 50x read depth of each bisulfite sequencing is shown as a dot on the curve. Among the targeted 84Mb covering ~3.7 million CpG sites (~13%) of human CpGs, more than 98.8% is covered and 50x read depth is more than 53% of target coverage. (H) Targeted bisulfite sequencing in DMSO or GSK343 treated SH-SY5Y cells. Scatter plots show the correlations of targeted bisulfite sequencing in DMSO or GSK343 treated cells as well as between biological duplicate samples. Pearson correlation coefficient is shown within each plot.

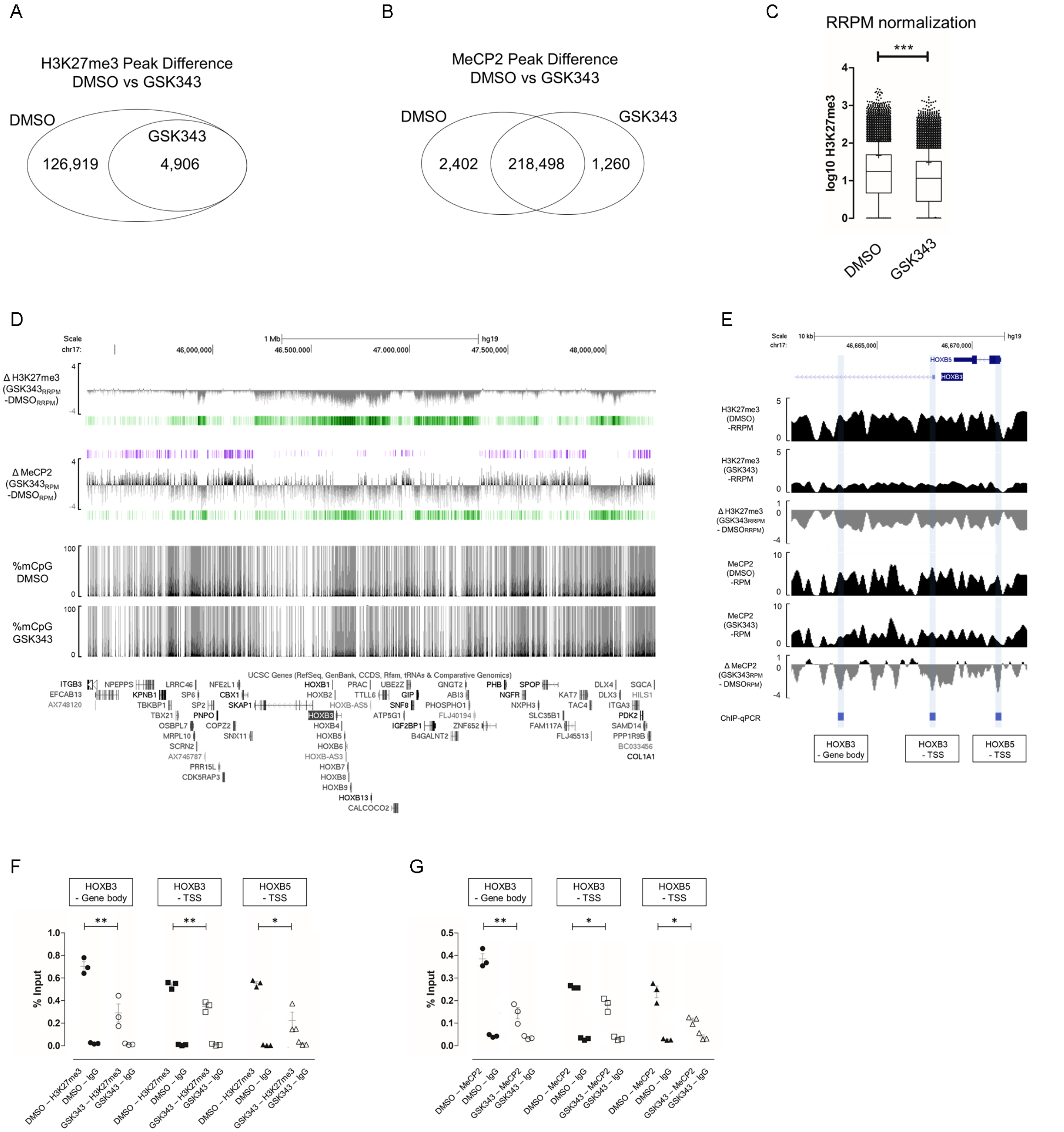


**Supplementary Fig. 7. GSK343 effect on H3K27me3 and MeCP2 binding correlation** (A and B) Venn diagram showing the number of common and unique H3K27me3 (A) or MeCP2 (B) peaks between DMSO- and GSK343 treated cells. (C) After normalization with RRPM, the distribution of H3K27me3 enrichment in gene body regions of DMSO- and GSK343 treated SH-SY5Y cells were plotted. N = 2 (per treatment) biologically independent experiments. Median H3K27me3 [log10] of DMSO = 1.25 and GSK343 = 1.06. $p < 0.00$ (Wilcoxon signed rank test, *** $p < 0.0001$). $p = 0.00$ were reported as $p < 0.0001$. Box-and-whisker plots show median, 10th and 90th percentile,

and min and max values. Mean is marked by "+." (D) Top two wiggle plots showing H3K27me3- and MeCP2 enrichment difference between DMSO- and GSK343 treated SH-SY5Y cells. Purple and green bars indicate increase and decrease of signal. Bottom two tracks illustrating the percentage of Methyl-C/C in either DMSO- or GSK343 treated SH-SY5Y. (E) Wiggle plots showing H3K27me3- and MeCP2 enrichment around the *HOXB* genes in both DMSO- and GSK343-treated SH-SY5Y cells. Blue bars indicate loci for ChIPed DNA quantification. (F) ChIP-qPCR showing average of three H3K27me3 ChIP / Input (%) in either DMSO- or GSK343- treated SH-SY5Y cells. N = 3 (per treatment, DMSO vs GSK343) biologically independent experiments. $p = 0.01$, $t_4 = 4.677$ (Genebody of *HOXB3*); $p = 0.004$, $t_4 = 5.963$ (TSS of *HOXB3*); $p = 0.013$, $t_4 = 4.291$ (TSS of *HOXB5*). (G) Same plot as in (F), for MeCP2 ChIP / Input (%). $p = 0.002$, $t_4 = 6.882$ (Genebody of *HOXB3*); $p = 0.013$, $t_4 = 4.256$ (TSS of *HOXB3*); $p = 0.011$, $t_4 = 4.544$ (TSS of *HOXB5*). (Two-tailed t-test, * $p < 0.05$, and ** $p < 0.01$). Graphs with individual data show average ± s.e.m.

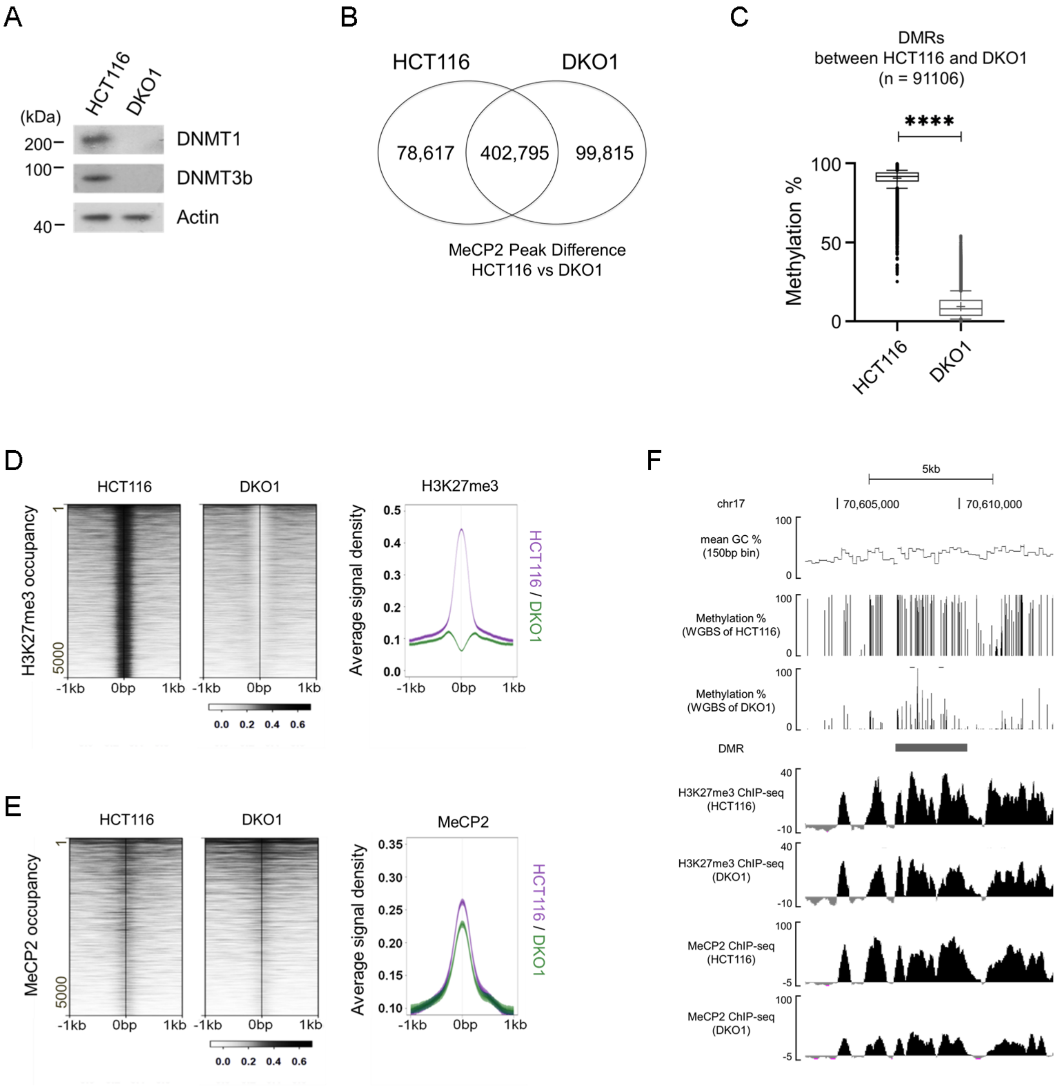


**Supplementary Fig. 8. H3K27me3 dependent MeCP2 binding in hypomethylation cells.** (A) Western blot showing absence of DNMT1 and DNMT3B in DKO1. Whole cell extracts were separated by SDS page and immune-blotted with anti-DNMT1, and DNMT3b. Anti-actin used as loading controls. Source data are provided as a Source Data file. (B) Venn diagram showing the number of common and unique MeCP2 peaks between HCT116 or DKO1 cells. (C) DNA methylation difference between HCT116 and DKO1 at DMRs. N = 1 (per genotype, HCT116 vs DKO1). n = 91,106, $p < 0.0001$ (two-tailed Wilcoxon signed-rank test, *** $p < 0.0001$). Box-and-whisker plots show median, 10th and 90th percentile, and min and max values. Mean is marked by "+." p = 0.00 were reported as $p < 0.0001$. (D) Heatmap illustrating H3K27me3 occupancy difference between HCT116 and DKO1 cells. H3K27me3 enrichment shown in either HCT116 (left panel) or DKO1 (center panel). Peaks are sorted according to signal intensity. Averaged H3K27me3 signals are given for HCT116 (purple) and DKO1 (green). (E) Heatmap showing changes in MeCP2 occupancy at the same loci of (E). Peaks are sorted according to signal intensity. Averaged MeCP2 signals are given for HCT116 (purple) and DKO1 (green). (F) Representative example of MeCP2 binding changes in H3K27me3 decreased loci of DKO1. UCSC genome-browser view (hg19) on chr17: 70,605,000 – 70,613,000.

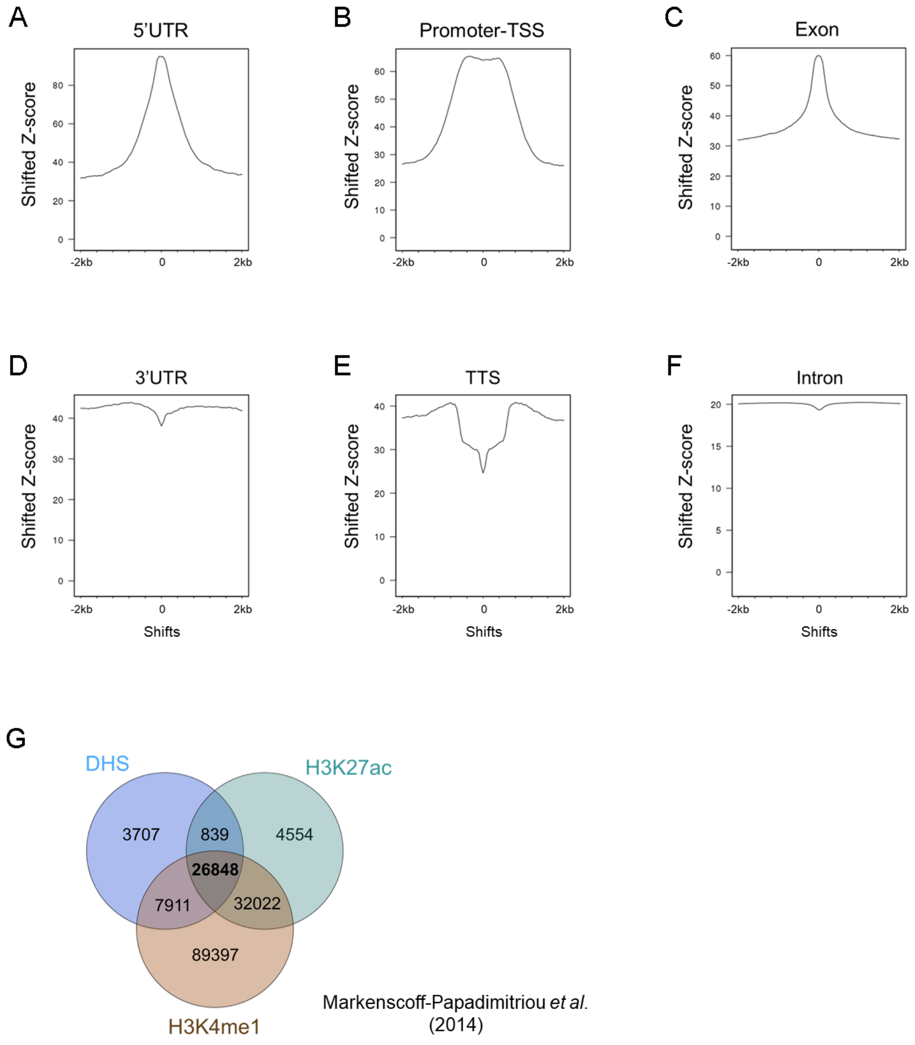


**Supplementary Fig. 9. Local Z-score analysis of MeCP2 binding in annotated gene structure regions.** (A) Local Z-score of 5'UTR (2-Kb window), (B) Promoter-TSS, (C) Exon, (D) 3'UTR, (E) TTS, and (F) Intron. (A, B, and C) The distribution of MeCP2 binding Z-score display as a narrow peak at the loci which dropped sharply when the MeCP2 peak shifted by 500 bp in either direction, suggesting that the association was highly dependent on their exact location. (D, E, and F) In 3'UTR, TTS, and intron regions, the distribution of Z-scores did not decrease when the MeCP2 peak shifted in either direction, indicating that the association of MeCP2 was not strictly dependent on the exact location of the peak. (G) Venn diagram showing overlaps between DHSs (DNase I hypersensitive sites), H3K4me1 and H3K27ac peaks. Referenced by previous studies[4], loci of DHS+ / H3K4me1+ / H3K27ac+ are defined as OE specific enhancer.

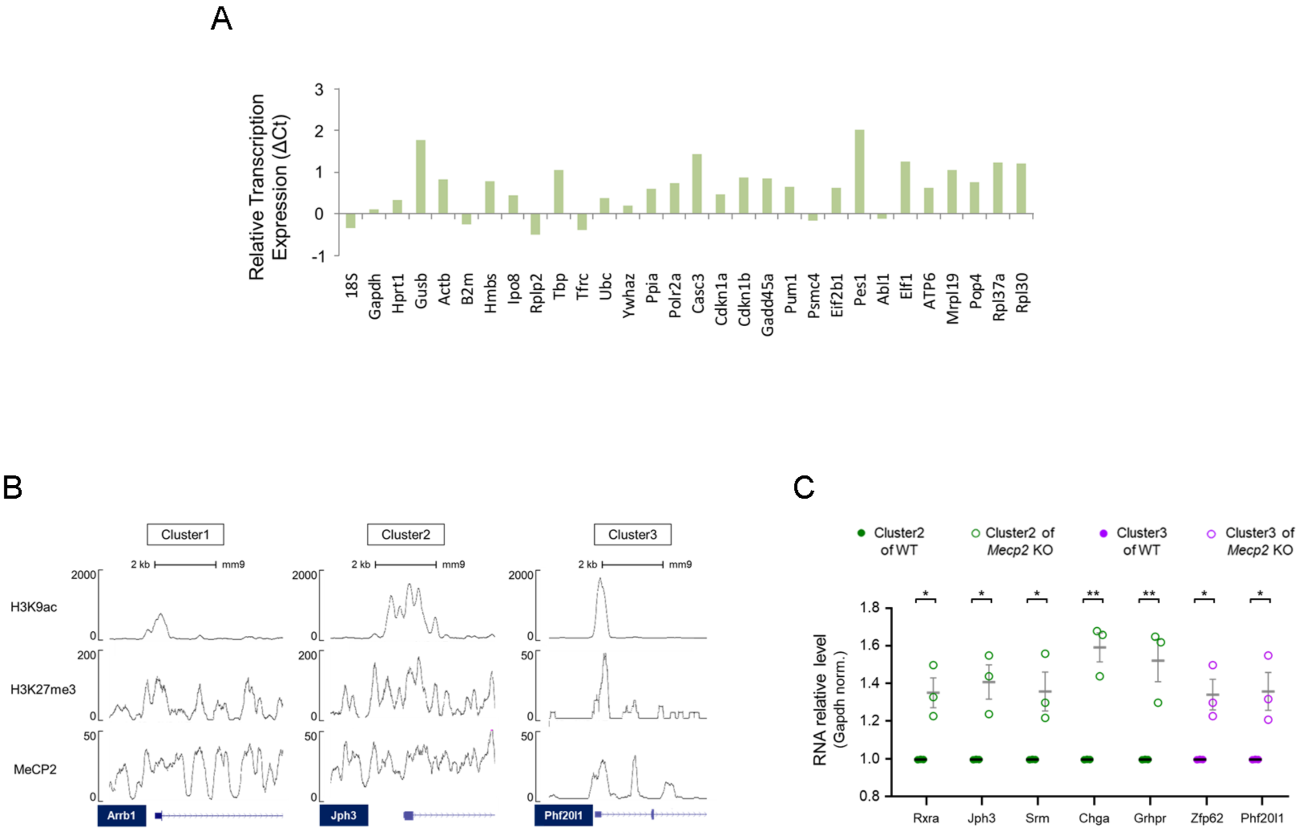


**Supplementary Fig. 10. Differential gene expression regulation depending on H3K27me3 and H3K9ac in *Mecp2* KO.** (A) Taqman qRT-PCR analysis of a panel of endogenous genes. Fold changes between *Mecp2* KO and WT OE were determined by ddCt method. ΔCt > 0 indicates decreased expression in *Mecp2* KO compared to WT, and *vice versa*. Notably, with the exception of *Gapdh*, *B2m*, and *Abl1*, most genes commonly used for gene expression normalization were over- or under-expressed 1.5- to 2-fold. (B) Genome browser views of comprehensive epigenetic modifications to an example gene belonging to cluster 1, cluster 2, and cluster 3. (C) RT-qPCR experiments are plotted in a box plot demonstrating fold-change of gene expression between WT and *Mecp2* KO using RT-qPCR. N = 3 (per genotype, WT vs *Mecp2* KO) from biologically independent mice. p = 0.011, $t_4$ = 4.483 (*Rxra*); p = 0.011, $t_4$ = 4.518 (*Jph3*); p = 0.025, $t_4$ = 3.508 (*Srm*); p = 0.002, $t_4$ = 7.717 (*Chga*); p = 0.009, $t_4$ = 4.672 (*Grhpr*); p = 0.013, $t_4$ = 4.244 (*Zfp62*); p = 0.023, $t_4$ = 3.594 (*Phf201l*) (Two-tailed t-test, * p < 0.05, and ** p < 0.01). Graphs with individual data show average ± s.e.m.

**Supplementary Table 1. Summary of ChIP-seq sequencing data**

| ChIP-seq in Olfactory Epithelium | | | | | | | | | | | | | | |
|---|---|---|---|---|---|---|---|---|---|---|---|---|---|---|
| Sample # | Accession | Title | Modification Profiled | Organism | Cell-Type | Read Length | Ref. | Total reads | Overall allignment rate | aligned 0 times | aligned exactly 1time | aligned > 1times | Unique Reads | Duplicated Reads |
| 1 | GSM1827604 | Input_WT_OE_rep1 | Input | Mus Musculus | Olfactory Epithelium | 50 | mm9 | 119900968 | 81.37% | 22337881 (18.63%) | 67267669 (56.10%) | 30295418 (25.27%) | 78,956,742 | 18,606,345 |
| 2 | GSM1827605 | Input_WT_OE_rep2 | Input | Mus Musculus | Olfactory Epithelium | 50 | mm9 | 96389916 | 93.34% | 6423402 (6.66%) | 64752232 (67.18%) | 25214282 (26.16%) | 56,390,363 | 33,576,151 |
| 3 | GSM1827606 | MeCP2_ChIP_OE_rep1 | MeCP2 | Mus Musculus | Olfactory Epithelium | 50 | mm9 | 455023339 | 81.20% | 85524197 (18.80%) | 285797132 (62.81%) | 83702010 (18.40%) | 87,353,667 | 282,145,475 |
| 4 | GSM1827607 | MeCP2_ChIP_OE_rep2 | MeCP2 | Mus Musculus | Olfactory Epithelium | 50 | mm9 | 220736216 | 88.63% | 25092455 (11.37%) | 150743525 (68.29%) | 44900236 (20.34%) | 97,407,847 | 98,235,914 |
| 5 | GSM1827608 | Mnase-seq | Mnase | Mus Musculus | Olfactory Epithelium | 50 | mm9 | 206792372 | 88.58% | 23615028 (11.42%) | 105199043 (50.87%) | 77978301 (37.71%) | 120,746,446 | 62,430,898 |
| 6 | GSM3465057 | HistoneH1_ChIP_OE_rep1 | Histone H1 | Mus Musculus | Olfactory Epithelium | 50 | mm9 | 20300649 | 92.31% | 1560310 (7.69%) | 13451792 (66.26%) | 5288547 (26.05%) | 14,753,497 | 3,986,842 |
| 7 | GSM3465058 | HistoneH1_ChIP_OE_rep2 | Histone H1 | Mus Musculus | Olfactory Epithelium | 50 | mm9 | 25706259 | 91.60% | 2159851 (8.40%) | 17130833 (66.64%) | 6415575 (24.96%) | 17,109,317 | 6,437,091 |
| 8 | GSM3465061 | H3K27me3_ChIP_OE_rep1 | H3K27me3 | Mus Musculus | Olfactory Epithelium | 50 | mm9 | 12984854 | 86.52% | 2452456 (13.48%) | 11412993 (62.71%) | 4332990 (23.81%) | 14,090,635 | 1,655,348 |
| 9 | GSM3465062 | H3K27me3_ChIP_OE_rep2 | H3K27me3 | Mus Musculus | Olfactory Epithelium | 50 | mm9 | 18198439 | 87.48% | 1625178 (12.52%) | 8340859 (64.24%) | 3018817 (23.25%) | 10,282,148 | 1,077,528 |
| 10 | GSM3465059 | H3K9ac_ChIP_OE_rep1 | H3K9ac | Mus Musculus | Olfactory Epithelium | 50 | mm9 | 19071623 | 85.41% | 2782107 (14.59%) | 13973079 (73.27%) | 2316437 (12.15%) | 11,528,100 | 4,761,416 |
| 11 | GSM3465060 | H3K9ac_ChIP_OE_rep2 | H3K9ac | Mus Musculus | Olfactory Epithelium | 50 | mm9 | 19506477 | 87.54% | 2430778 (12.46%) | 14787401 (75.81%) | 2288298 (11.73%) | 13,028,992 | 4,046,707 |

| ChIP-Rx in SH-SY5Y (DMSO vs GSK343) | | | | | | | | | | | | | | |
|---|---|---|---|---|---|---|---|---|---|---|---|---|---|---|
| Sample # | Accession | Title | Modification Profiled | Organism | Cell-Type | Read Length | Ref. | Total reads | Overall allignment rate | aligned 0 times | aligned exactly 1time | aligned > 1times | Unique Reads | Duplicated Reads |
| 12 | GSM3465063 | Input_ChIPRx_DMSO_rep1 | Input | Homo sapiens + Drosophila melanogaster | SH-SY5Y | 75 | hg19 | 25359778 | 91.42% | 2175749 (8.58%) | 19576271 (77.19%) | 3607758 (14.23%) | 21,833,667 | 1,350,362 |
| | | | | | | | dm3 | 25359778 | 3.68% | 24427151 (96.32%) | 694478 (2.74%) | 238149 (0.94%) | 837,493 | 95,134 |
| 13 | GSM3465064 | Input_ChIPRx_DMSO_rep2 | Input | Homo sapiens + Drosophila melanogaster | SH-SY5Y | 75 | hg19 | 28329416 | 90.89% | 2581325 (9.11%) | 21729584 (76.70%) | 4018507 (14.18%) | 24,264,441 | 1,483,650 |
| | | | | | | | dm3 | 28329416 | 4.02% | 27190477 (95.98%) | 845098 (2.98%) | 293841 (1.04%) | 1,016,961 | 121,978 |
| 14 | GSM3465067 | H3K27me3_ChIPRx_DMSO_rep1 | H3K27me3 | Homo sapiens + Drosophila melanogaster | SH-SY5Y | 75 | hg19 | 58755560 | 69.85% | 17716230 (30.15%) | 32885928 (55.97%) | 8153402 (13.88%) | 36,624,921 | 4,414,409 |
| | | | | | | | dm3 | 58755560 | 24.26% | 44504307 (75.74%) | 11661537 (19.85%) | 2589716 (4.41%) | 11,693,134 | 2,558,119 |
| 15 | GSM3465068 | H3K27me3_ChIPRx_DMSO_rep2 | H3K27me3 | Homo sapiens + Drosophila melanogaster | SH-SY5Y | 75 | hg19 | 56731025 | 69.84% | 17109940 (30.16%) | 31697162 (55.87%) | 7923923 (13.97%) | 35,772,491 | 3,848,594 |
| | | | | | | | dm3 | 56731025 | 24.30% | 42944032 (75.70%) | 11291741 (19.90%) | 2495252 (4.40%) | 11,444,175 | 2,342,818 |
| 16 | GSM3489380 | MeCP2_ChIPRx_DMSO_rep1 | MeCP2 | Homo sapiens + Drosophila melanogaster | SH-SY5Y | 75 | hg19 | 56046501 | 85.49% | 8129996 (14.51%) | 38123349 (68.02%) | 9793156 (17.47%) | 40,737,404 | 7,179,101 |
| | | | | | | | dm3 | 56046501 | 8.89% | 51065524 (91.11%) | 4169992 (7.44%) | 810985 (1.45%) | 4,060,742 | 920,235 |
| 17 | GSM3489381 | MeCP2_ChIPRx_DMSO_rep2 | MeCP2 | Homo sapiens + Drosophila melanogaster | SH-SY5Y | 75 | hg19 | 57359128 | 85.53% | 8297174 (14.47%) | 39058731 (68.10%) | 10003223 (17.44%) | 39,350,269 | 9,711,685 |
| | | | | | | | dm3 | 57359128 | 8.79% | 52317015 (91.21%) | 4223269 (7.36%) | 818844 (1.43%) | 3,878,975 | 1,163,138 |
| 18 | GSM3465065 | Input_ChIPRx_GSK343_rep1 | Input | Homo sapiens + Drosophila melanogaster | SH-SY5Y | 75 | hg19 | 24001928 | 91.30% | 2088403 (8.70%) | 18498553 (77.07%) | 3414972 (14.23%) | 20,776,516 | 1,137,009 |
| | | | | | | | dm3 | 24001928 | 3.72% | 23107935 (96.28%) | 660526 (2.75%) | 233467 (0.97%) | 798,100 | 95,893 |
| 19 | GSM3465066 | Input_ChIPRx_GSK343_rep2 | Input | Homo sapiens + Drosophila melanogaster | SH-SY5Y | 75 | hg19 | 26406300 | 90.97% | 2385172 (9.03%) | 20305504 (76.90%) | 3715624 (14.07%) | 22,621,480 | 1,399,648 |
| | | | | | | | dm3 | 26406300 | 4.04% | 25340652 (95.96%) | 788819 (2.99%) | 276829 (1.05%) | 940,486 | 125,162 |

| | | | | | | | | | | | | | | |
|---|---|---|---|---|---|---|---|---|---|---|---|---|---|---|
| 20 | GSM3465069 | H3K27me3_ChIPRx_GSK343_rep1 | H3K27me3 | Homo sapiens + Drosophila melanogaster | SH-SY5Y | 75 | hg19 | 52602478 | 59.34% | 21389751 (40.66%) | 25007576 (47.54%) | 6205151 (11.80%) | 28,745,582 | 2,467,145 |
| | | | | | | | dm3 | 52602478 | 33.99% | 34722672 (66.01%) | 14522241 (27.61%) | 3357565 (6.38%) | 14,643,962 | 3,235,844 |
| 21 | GSM3465070 | H3K27me3_ChIPRx_GSK343_rep2 | H3K27me3 | Homo sapiens + Drosophila melanogaster | SH-SY5Y | 75 | hg19 | 53044967 | 59.13% | 21679479 (40.87%) | 25090092 (47.30%) | 6275396 (11.83%) | 28,436,967 | 2,928,521 |
| | | | | | | | dm3 | 53044967 | 34.61% | 34684095 (65.39%) | 14950824 (28.19%) | 3410048 (6.43%) | 14,815,385 | 3,545,487 |
| 22 | GSM3489382 | MeCP2_ChIPRx_GSK343_rep1 | MeCP2 | Homo sapiens + Drosophila melanogaster | SH-SY5Y | 75 | hg19 | 52397604 | 87.01% | 6806250 (12.99%) | 36482937 (69.63%) | 9108417 (17.38%) | 34,904,562 | 6,879,155 |
| | | | | | | | dm3 | 52397604 | 7.11% | 48673053 (92.89%) | 3118878 (5.95%) | 605673 (1.16%) | 2,978,556 | 745,995 |
| 23 | GSM3489383 | MeCP2_ChIPRx_GSK343_rep2 | MeCP2 | Homo sapiens + Drosophila melanogaster | SH-SY5Y | 75 | hg19 | 53027789 | 86.77% | 7017285 (13.23%) | 36752530 (69.31%) | 9257974 (17.46%) | 35,318,873 | 10,691,631 |
| | | | | | | | dm3 | 53027789 | 7.05% | 49288616 (92.95%) | 3127608 (5.90%) | 611565 (1.15%) | 2,792,716 | 946,457 |

**ChIP-Rx in HCT116 (WT vs DKO1)**

| Sample # | Accession | Title | Modification Profiled | Organism | Cell-Type | Read Length | Ref. | Total reads | Overall allignment rate | aligned 0 times | aligned exactly 1time | aligned > 1times | Unique Reads | Duplicated Reads |
|---|---|---|---|---|---|---|---|---|---|---|---|---|---|---|
| 24 | GSM4041342 | Input_ChIPRx_HCT116_rep1 | Input | Homo sapiens + Drosophila melanogaster | HCT116 | 75 | hg19 | 40575403 | 93.33% | 2706844 (6.67%) | 33005085 (81.34%) | 4863474 (11.99%) | 35,359,997 | 2,508,562 |
| | | | | | | | dm3 | 40575403 | 2.12% | 39714597 (97.88%) | 596589 (1.47%) | 264217 (0.65%) | 735,589 | 125,217 |
| 25 | GSM4041343 | Input_ChIPRx_HCT116_rep2 | Input | Homo sapiens + Drosophila melanogaster | HCT116 | 75 | hg19 | 38316825 | 93.11% | 2639605 (6.89%) | 31106451 (81.18%) | 4570769 (11.93%) | 33,567,921 | 2,109,299 |
| | | | | | | | dm3 | 38316825 | 2.17% | 37486059 (97.83%) | 581983 (1.52%) | 248783 (0.65%) | 716,684 | 114,082 |
| 26 | GSM4041346 | H3K27me3_ChIPRx_HCT116_rep1 | H3K27me3 | Homo sapiens + Drosophila melanogaster | HCT116 | 75 | hg19 | 85769374 | 83.79% | 13902001 (16.21%) | 51728787 (60.31%) | 20138586 (23.48%) | 21,150,550 | 50,716,823 |
| | | | | | | | dm3 | 85769374 | 13.64% | 74072168 (86.36%) | 9386258 (10.94%) | 2310948 (2.69%) | 3,575,893 | 8,121,313 |
| 27 | GSM4041347 | H3K27me3_ChIPRx_HCT116_rep2 | H3K27me3 | Homo sapiens + Drosophila melanogaster | HCT116 | 75 | hg19 | 85280805 | 88.59% | 9731269 (11.41%) | 54095674 (63.43%) | 21453862 (25.16%) | 39,010,684 | 36,538,852 |
| | | | | | | | dm3 | 85280805 | 9.02% | 77590008 (90.98%) | 6241690 (7.32%) | 1449107 (1.70%) | 3,953,157 | 3,737,640 |
| 28 | GSM4041350 | MeCP2_ChIPRx_HCT116_rep1 | MeCP2 | Homo sapiens + Drosophila melanogaster | HCT116 | 75 | hg19 | 87629874 | 74.70% | 22167826 (25.30%) | 46345955 (52.89%) | 19116093 (21.81%) | 40,133,788 | 25,328,260 |
| | | | | | | | dm3 | 87629874 | 22.54% | 67875152 (77.46%) | 15439672 (17.62%) | 4315050 (4.92%) | 11,055,003 | 8,699,719 |
| 29 | GSM4041351 | MeCP2_ChIPRx_HCT116_rep2 | MeCP2 | Homo sapiens + Drosophila melanogaster | HCT116 | 75 | hg19 | 74583545 | 75.40% | 18346123 (24.60%) | 39549999 (53.03%) | 16687423 (22.37%) | 39,271,036 | 16,966,386 |
| | | | | | | | dm3 | 74583545 | 21.82% | 58311072 (78.18%) | 12794113 (17.15%) | 3478360 (4.66%) | 10,390,980 | 5,881,493 |
| 30 | GSM4041344 | Input_ChIPRx_DKO1_rep1 | Input | Homo sapiens + Drosophila melanogaster | DKO1 | 75 | hg19 | 38244012 | 93.74% | 2393971 (6.26%) | 31239193 (81.68%) | 4610848 (12.06%) | 33,703,191 | 2,146,850 |
| | | | | | | | dm3 | 38244012 | 1.80% | 37555860 (98.20%) | 479343 (1.25%) | 208809 (0.55%) | 589,111 | 99,041 |
| 31 | GSM4041345 | Input_ChIPRx_DKO1_rep2 | Input | Homo sapiens + Drosophila melanogaster | DKO1 | 75 | hg19 | 41230577 | 93.39% | 2726228 (6.61%) | 33580211 (81.44%) | 4924138 (11.94%) | 36,123,010 | 2,381,339 |
| | | | | | | | dm3 | 41230577 | 1.87% | 40459832 (98.13%) | 541203 (1.31%) | 229542 (0.56%) | 650,203 | 120,542 |
| 32 | GSM4041348 | H3K27me3_ChIPRx_DKO1_rep1 | H3K27me3 | Homo sapiens + Drosophila melanogaster | DKO1 | 75 | hg19 | 87310810 | 89.81% | 8893835 (10.19%) | 56667996 (64.90%) | 21748979 (24.91%) | 31,012,096 | 47,404,879 |
| | | | | | | | dm3 | 87310810 | 7.99% | 80333891 (92.01%) | 5718379 (6.55%) | 1258540 (1.44%) | 2,825,733 | 4,151,186 |
| 33 | GSM4041349 | H3K27me3_ChIPRx_DKO1_rep2 | H3K27me3 | Homo sapiens + Drosophila melanogaster | DKO1 | 75 | hg19 | 73081755 | 91.84% | 5960622 (8.16%) | 48456911 (66.31%) | 18664222 (25.54%) | 40,714,617 | 26,406,516 |
| | | | | | | | dm3 | 73081755 | 6.00% | 68699113 (94.00%) | 3588994 (4.91%) | 793648 (1.09%) | 2,643,020 | 1,739,622 |
| 34 | GSM4041352 | MeCP2_ChIPRx_DKO1_rep1 | MeCP2 | Homo sapiens + Drosophila melanogaster | DKO1 | 75 | hg19 | 86501051 | 82.67% | 14987520 (17.33%) | 50738892 (58.66%) | 20774639 (24.02%) | 52,802,550 | 18,710,981 |
| | | | | | | | dm3 | 86501051 | 14.26% | 74167772 (85.74%) | 9746421 (11.27%) | 2586858 (2.99%) | 8,589,121 | 3,744,158 |

| | | | | | | | | | | | | | | |
|---|---|---|---|---|---|---|---|---|---|---|---|---|---|---|
| 35 | GSM4041353 | MeCP2_ChIPRx_DKO1_rep2 | MeCP2 | Homo sapiens + Drosophila melanogaster | DKO1 | 75 | hg19 | 73748889 | 83.33% | 12295607 (16.67%) | 43662955 (59.20%) | 17790327 (24.12%) | 42,657,362 | 18,795,920 |
| | | | | | | | dm3 | 73748889 | 13.55% | 63757299 (86.45%) | 7865591 (10.67%) | 2125999 (2.88%) | 6,635,798 | 3,355,792 |

**Supplementary Table 2. Summary of targeted bisulfite sequencing data**

| Targeted bisulfite sequencing in SH-SY5Y (DMSO vs GSK343) | | | | | | | | | | | | | |
|---|---|---|---|---|---|---|---|---|---|---|---|---|---|
| Sample # | Accession | Title | Treatment | Organism | Cell-Type | Read Length | Ref. | Total reads | Aligned reads | Unaligned reads | Ambiguously aligned reads | Unique Reads | Duplicated Reads |
| 1 | GSM4041354 | DMSO_re1 | DMSO | Homo sapiens | SH-SY5Y | 101 | hg19 | 47,971,907 | 38,475,795 | 8,865,086 | 631,019 | 34,764,359 | 3,711,436 |
| 2 | GSM4041355 | DMSO_re2 | DMSO | Homo sapiens | SH-SY5Y | 101 | hg19 | 50,486,128 | 40,183,061 | 9,634,124 | 668,932 | 36,123,494 | 4,059,567 |
| 3 | GSM4041356 | GSK343_re1 | GSK343 | Homo sapiens | SH-SY5Y | 101 | hg19 | 49,202,772 | 39,526,626 | 9,059,511 | 616,627 | 35,539,692 | 3,986,934 |
| 4 | GSM4041357 | GSK343_re2 | GSK343 | Homo sapiens | SH-SY5Y | 101 | hg19 | 50,208,850 | 40,197,248 | 9,356,803 | 654,798 | 36,172,386 | 4,024,862 |

**Supplementary Table 3. Datasets for exogenous reference derived normalization factors.**

| ChIP-Rx in SH-SY5Y (DMSO vs GSK343) | | | | | | | | | | | | |
|---|---|---|---|---|---|---|---|---|---|---|---|---|
| **Accession** | **Title** | **Modification Profiled** | **Organism** | **Cell-Type** | **Ratio (Human:Dros.)** | **Read Length** | **Reads aligning to Human** | **Reads aligning to Drosophila** | **Nh†** | **Scale factor of RPM (α††)** | **Nd*** | **Scale factor of RRPM (α-x**)** |
| GSM3465063 + GSM3465064 | Input_ChIPRx_DMSO_Merge | Input | Homo sapiens + Drosophila melanogaster | SH-SY5Y | 3:1 | 75 | 46,098,108 | 1,854,454 | 46.098 | 0.217 | 1.854 | 0.539 |
| GSM3465067 + GSM3465068 | H3K27me3_ChIPRx_DMSO_Merge | H3K27me3 | Homo sapiens + Drosophila melanogaster | SH-SY5Y | 3:1 | 75 | 72,397,412 | 23,137,309 | 72.397 | 0.138 | 23.137 | **0.043** |
| GSM3489380 + GSM3489381 | MeCP2_ChIPRx_DMSO_Merge | MeCP2 | Homo sapiens + Drosophila melanogaster | SH-SY5Y | 3:1 | 75 | 80,087,673 | 7,939,717 | 80.088 | **0.125** | 7.940 | 0.126 |
| GSM3465065 + GSM3465066 | Input_ChIPRx_GSK343_Merge | Input | Homo sapiens + Drosophila melanogaster | SH-SY5Y | 3:1 | 75 | 43,397,996 | 1,738,586 | 43.398 | 0.230 | 1.739 | 0.575 |
| GSM3465069 + GSM3465070 | H3K27me3_ChIPRx_GSK343_Merge | H3K27me3 | Homo sapiens + Drosophila melanogaster | SH-SY5Y | 3:1 | 75 | 57,182,549 | 29,459,347 | 57.183 | 0.175 | 29.459 | **0.034** |
| GSM3489382 + GSM3489383 | MeCP2_ChIPRx_GSK343_Merge | MeCP2 | Homo sapiens + Drosophila melanogaster | SH-SY5Y | 3:1 | 75 | 70,223,435 | 5,771,272 | 70.223 | **0.142** | 5.771 | 0.173 |

Nh† = The number of uniquely aligned reads to the Homo sapiens (hg19) per millions

α†† = Scale factor for RPM, 10 / Nh†

Nd* = The number of uniquely aligned reads to the Dros.(dm3) per millions

α-x** = Scale factor for RRPM,  1 / Nd*

The scaling factors for normalization for each analsys are shown in bold.

**Supplementary Table 4. List of genes in each cluster of NanoString panel**

| Group | Probe Name | Accession# | Nanostring Panel | NS Probe ID | log2(Avg_WT) | log2(Avg_KO) | log2(KO/WT) |
|---|---|---|---|---|---|---|---|
| Cluster1 | Pvalb | NM_013645.3 | Neuropathology | NM_013645.3:60 | 4.865918815 | 2.95419631 | -1.911722504 |
| Cluster1 | Slc32a1 | NM_009508.2 | Neuropathology | NM_009508.2:2616 | 6.04843238 | 4.190219764 | -1.858212617 |
| Cluster1 | Dbh | NM_138942.3 | Neuropathology | NM_138942.3:254 | 2.9800253 | 1.577730931 | -1.402294369 |
| Cluster1 | Camk2b | NM_001174053.1 | Neuropathology | NM_001174053.1:2825 | 6.882643049 | 5.499686464 | -1.382956585 |
| Cluster1 | Grm2 | NM_001160353.1 | Neuropathology | NM_001160353.1:2770 | 4.913846824 | 3.713695815 | -1.200151009 |
| Cluster1 | Th | NM_009377.1 | Neuropathology | NM_009377.1:235 | 4.713695815 | 3.545350645 | -1.16834517 |
| Cluster1 | Cacna1s | NM_014193.2 | Neuropathology | NM_014193.2:3465 | 3.517905554 | 2.509695842 | -1.008209712 |
| Cluster1 | Dlx2 | NM_010054.2 | Neuropathology | NM_010054.2:1891 | 3.819668183 | 2.95419631 | -0.865471873 |
| Cluster1 | Pla2g2f | NM_012045.4 | Neuropathology | NM_012045.4:1802 | 3.885574364 | 3.030336078 | -0.855238286 |
| Cluster1 | Gfap | NM_001131020.1 | Neuropathology | NM_001131020.1:610 | 5.443606651 | 4.601399391 | -0.842207261 |
| Cluster1 | Cck | NM_031161.2 | Neuropathology | NM_031161.2:351 | 5.418527302 | 4.593353771 | -0.825173531 |
| Cluster1 | Grin2b | NM_008171.3 | Neuropathology | NM_008171.3:6340 | 4.987320866 | 4.190219764 | -0.797101102 |
| Cluster1 | Sorcs3 | NM_025696.3 | Neuropathology | NM_025696.3:1868 | 4.398829182 | 3.713695815 | -0.685133368 |
| Cluster1 | Prkcg | NM_011102.3 | Neuropathology | NM_011102.3:1580 | 4.508111681 | 3.910252963 | -0.597858718 |
| Cluster1 | Cplx1 | NM_007756.3 | Neuropathology | NM_007756.3:327 | 5.177917792 | 4.677508242 | -0.50040955 |
| Cluster1 | Adora1 | NM_001008533.3 | Neuropathology | NM_001008533.3:1603 | 4.089159132 | 3.590362488 | -0.498796643 |
| Cluster1 | Gabrb3 | NM_008071.3 | Neuropathology | NM_008071.3:4200 | 5.51080372 | 5.022367813 | -0.488435907 |
| Cluster1 | Trpv1 | NM_001001445.1 | Neuropathology | NM_001001445.1:1710 | 3.579542225 | 3.110196178 | -0.469346047 |
| Cluster1 | Lif | NM_008501.2 | Neuropathology | NM_008501.2:3435 | 4.823494625 | 4.357552005 | -0.46594262 |
| Cluster1 | Cntnap1 | NM_016782.2 | Neuropathology | NM_016782.2:1105 | 5.554588852 | 5.094869433 | -0.459719419 |
| Cluster1 | Stx1b | NM_024414.2 | Neuropathology | NM_024414.2:570 | 7.798536603 | 7.346646694 | -0.451889909 |
| Cluster1 | Sp100 | NM_013673.3 | Neuropathology | NM_013673.3:410 | 7.193771743 | 6.77774846 | -0.416023283 |
| Cluster1 | Thy1 | NM_009382.3 | Neuropathology | NM_009382.3:425 | 7.683731539 | 7.278449458 | -0.405282081 |
| Cluster1 | Tradd | NM_001033161.2 | Neuropathology | NM_001033161.2:562 | 4.789468437 | 4.389910925 | -0.399557512 |
| Cluster1 | Phf19 | NM_028716.4 | Neuropathology | NM_028716.4:735 | 3.957450336 | 3.56193706 | -0.395513277 |
| Cluster1 | Mag | NM_010758.2 | Neuropathology | NM_010758.2:1670 | 4.06091205 | 3.666756592 | -0.394155458 |
| Cluster1 | Cd4 | NM_013488.2 | Neuropathology | NM_013488.2:950 | 3.995032192 | 3.617651119 | -0.377381073 |
| Cluster1 | Pla2g4e | NM_177845.4 | Neuropathology | NM_177845.4:1016 | 3.475733431 | 3.10349764 | -0.372235791 |
| Cluster1 | Cacna1a | NM_007578.3 | Neuropathology | NM_007578.3:1655 | 7.585037639 | 7.225014583 | -0.360023057 |
| Cluster1 | Slc6a4 | NM_010484.2 | Neuropathology | NM_010484.2:2200 | 4.068240861 | 3.713695815 | -0.354545046 |
| Cluster1 | Drd4 | NM_007878.2 | Neuropathology | NM_007878.2:962 | 3.442943496 | 3.095080492 | -0.347863004 |
| Cluster1 | Fa2h | NM_178086.3 | Neuropathology | NM_178086.3:644 | 3.376429311 | 3.030336078 | -0.346093233 |
| Cluster1 | Egr1 | NM_007913.5 | Neuropathology | NM_007913.5:515 | 6.833902077 | 6.520343668 | -0.313558408 |
| Cluster1 | Bdnf | NM_007540.4 | Neuropathology | NM_007540.4:3260 | 4.638653116 | 4.330916878 | -0.307736238 |
| Cluster1 | Atp6v1g2 | NM_023179.3 | Neuropathology | NM_023179.3:994 | 6.626877135 | 6.329213313 | -0.297663822 |
| Cluster1 | Pdgfrb | NM_008809.1 | Neuropathology | NM_008809.1:1185 | 6.570007463 | 6.272863023 | -0.29714444 |
| Cluster1 | C1qa | NM_007572.2 | Neuropathology | NM_007572.2:566 | 7.542567608 | 7.25219234 | -0.290375268 |
| Cluster1 | Prkcb | NM_008855.2 | Neuropathology | NM_008855.2:8332 | 6.795779933 | 6.506367093 | -0.28941284 |
| Cluster1 | Flt1 | NM_010228.3 | Neuropathology | NM_010228.3:1550 | 5.443606651 | 5.154818109 | -0.288788542 |
| Cluster1 | Islr2 | NM_001161538.1 | Neuropathology | NM_001161538.1:1782 | 4.685940148 | 4.409051439 | -0.27688871 |
| Cluster1 | Pla2g2e | NM_012044.2 | Neuropathology | NM_012044.2:224 | 2.454175893 | 2.177917792 | -0.276258101 |
| Cluster1 | Csf1 | NM_001113530.1 | Neuropathology | NM_001113530.1:833 | 7.595518579 | 7.320800549 | -0.27471803 |
| Cluster1 | Efnb3 | NM_007911.5 | Neuropathology | NM_007911.5:2880 | 7.220765252 | 6.947315492 | -0.27344976 |
| Cluster1 | Htr5a | NM_008314.2 | Neuropathology | NM_008314.2:1696 | 3.74092756 | 3.467931546 | -0.272996014 |
| Cluster1 | Slc6a3 | NM_010020.3 | Neuropathology | NM_010020.3:1082 | 3.376429311 | 3.133399125 | -0.243030186 |
| Cluster1 | Nes | NM_016701.3 | Neuropathology | NM_016701.3:2716 | 5.74092756 | 5.507160349 | -0.233767211 |
| Cluster1 | Ager | NM_007425.2 | Neuropathology | NM_007425.2:361 | 4.476057596 | 4.252097703 | -0.223959893 |
| Cluster1 | Icam2 | NM_010494.1 | Neuropathology | NM_010494.1:375 | 5.676803354 | 5.454340425 | -0.222462929 |
| Cluster1 | Pkn1 | NM_001199593.1 | Neuropathology | NM_001199593.1:1380 | 6.875595783 | 6.653418353 | -0.222177431 |
| Cluster1 | Pla2g4d | NM_001024137.1 | Neuropathology | NM_001024137.1:2798 | 2.657640005 | 2.438292852 | -0.219347154 |
| Cluster1 | Bid | NM_007544.3 | Neuropathology | NM_007544.3:1307 | 5.921840937 | 5.705977902 | -0.215863035 |
| Cluster1 | Syt13 | NM_030725.4 | Neuropathology | NM_030725.4:2290 | 4.910013053 | 4.721372659 | -0.188640394 |
| Cluster1 | Hap1 | NM_010404.3 | Neuropathology | NM_010404.3:2552 | 7.186510462 | 7.011842227 | -0.174668235 |
| Cluster1 | Hspb1 | NM_013560.2 | Neuropathology | NM_013560.2:630 | 9.205402288 | 9.039261794 | -0.166140494 |
| Cluster1 | Avp | NM_009732.2 | Neuropathology | NM_009732.2:52 | 4.443606651 | 4.279842694 | -0.163763958 |
| Cluster1 | Gdnf | NM_010275.2 | Neuropathology | NM_010275.2:460 | 3.666756592 | 3.509695842 | -0.15706075 |
| Cluster1 | Tmem119 | NM_146162.2 | Neuropathology | NM_146162.2:1550 | 8.778060698 | 8.6383273 | -0.139733397 |
| Cluster1 | Cspg4 | NM_139001.2 | Neuropathology | NM_139001.2:1530 | 6.557348818 | 6.418527302 | -0.138821515 |
| Cluster1 | Nefl | NM_010910.1 | Neuropathology | NM_010910.1:1303 | 3.138323004 | 3.006298024 | -0.13202498 |
| Cluster1 | Plcb2 | NM_177568.2 | Neuropathology | NM_177568.2:4335 | 4.191799501 | 4.06307145 | -0.128728051 |
| Cluster1 | Trpm2 | NM_138301.2 | Neuropathology | NM_138301.2:2106 | 4.643567622 | 4.518849829 | -0.124717793 |
| Cluster1 | Npy | NM_023456.2 | Neuropathology | NM_023456.2:230 | 7.391157648 | 7.273282757 | -0.117874891 |
| Cluster1 | Cpt1b | NM_009948.2 | Neuropathology | NM_009948.2:924 | 4.454504938 | 4.337354298 | -0.117150639 |
| Cluster1 | Tcirg1 | NM_001136091.1 | Neuropathology | NM_001136091.1:1345 | 6.860155835 | 6.751142325 | -0.10901351 |
| Cluster1 | Slc18a2 | NM_172523.3 | Neuropathology | NM_172523.3:3160 | 3.750070486 | 3.650764559 | -0.099305927 |
| Cluster1 | C1qc | NM_007574.2 | Neuropathology | NM_007574.2:708 | 7.697766871 | 7.599950025 | -0.097816846 |
| Cluster1 | Cd40 | NM_011611.2 | Neuropathology | NM_011611.2:1425 | 3.723012396 | 3.634593268 | -0.088419128 |
| Cluster1 | Nos1 | NM_008712.2 | Neuropathology | NM_008712.2:2985 | 5.462379572 | 5.376950171 | -0.085429401 |
| Cluster1 | Nr4a2 | NM_001139509.1 | Neuropathology | NM_001139509.1:1626 | 6.478810059 | 6.399598369 | -0.07921169 |
| Cluster1 | Abat | NM_001170978.1 | Neuropathology | NM_001170978.1:3560 | 7.927096446 | 7.849061003 | -0.078035444 |
| Cluster1 | Lrrc25 | NM_153074.3 | Neuropathology | NM_153074.3:246 | 5.174925683 | 5.097189387 | -0.077736295 |
| Cluster1 | Cldn15 | NM_021719.4 | Neuropathology | NM_021719.4:1184 | 3.836429131 | 3.759155834 | -0.077273297 |
| Cluster1 | Slc12a5 | NM_020333.2 | Neuropathology | NM_020333.2:5618 | 7.105279975 | 7.031108429 | -0.074171546 |
| Cluster1 | Ngfr | NM_033217.3 | Neuropathology | NM_033217.3:1995 | 3.267535798 | 3.200849575 | -0.066686223 |
| Cluster1 | Notch4 | NM_010929.2 | Neuropathology | NM_010929.2:2436 | 3.528571319 | 3.462052319 | -0.066519 |
| Cluster1 | Pla2g6 | NM_001199023.1 | Neuropathology | NM_001199023.1:768 | 7.351955117 | 7.303461128 | -0.048493989 |
| Cluster1 | Spi1 | NM_011355.1 | Neuropathology | NM_011355.1:200 | 6.801417366 | 6.7554885 | -0.045928866 |

| | | | | | | | |
|---|---|---|---|---|---|---|---|
| Cluster1 | Lrp1 | NM_008512.2 | Neuropathology | NM_008512.2:1310 | 10.19969075 | 10.16006679 | -0.039623953 |
| Cluster1 | Psmb8 | NM_010724.2 | Neuropathology | NM_010724.2:362 | 7.116707838 | 7.077723675 | -0.038984164 |
| Cluster1 | Stab1 | NM_138672.2 | Neuropathology | NM_138672.2:5890 | 6.448818377 | 6.416924167 | -0.03189421 |
| Cluster1 | Gal3st1 | NM_001177691.1 | Neuropathology | NM_001177691.1:1197 | 4.387500406 | 4.373648211 | -0.013852195 |
| Cluster1 | Lmna | NM_001002011.2 | Neuropathology | NM_001002011.2:1611 | 9.312372478 | 9.300970236 | -0.011402242 |
| Cluster1 | Icam1 | NM_010493.2 | Neuropathology | NM_010493.2:2195 | 5.49217366 | 5.483170853 | -0.009002806 |
| Cluster1 | Epha2 | NM_010139.2 | Neuropathology | NM_010139.2:1705 | 5.384567923 | 5.378511623 | -0.0060563 |
| Cluster1 | Cdkn1a | NM_007669.4 | Neuropathology | NM_007669.4:1670 | 8.874320537 | 8.869655529 | -0.004665009 |
| Cluster1 | Ntrk1 | NM_001033124.1 | Neuropathology | NM_001033124.1:1481 | 3.364572432 | 3.360364277 | -0.004208156 |
| Cluster1 | Arhgef10 | NM_001037736.1 | Neuropathology | NM_001037736.1:1105 | 6.619413011 | 6.623735202 | 0.004322191 |
| Cluster1 | Egr2 | NM_010118.2 | Neuropathology | NM_010118.2:1785 | 3.025028794 | 3.030336078 | 0.005307284 |
| Cluster1 | Gstp1 | NM_013541.1 | Neuropathology | NM_013541.1:421 | 10.15752379 | 10.16666524 | 0.009141452 |
| Cluster1 | Npas4 | NM_153553.4 | Neuropathology | NM_153553.4:580 | 4.286511558 | 4.296824459 | 0.010312901 |
| Cluster1 | Egfr | NM_207655.2 | Neuropathology | NM_207655.2:1335 | 7.200408226 | 7.211012193 | 0.010603967 |
| Cluster1 | Tardbp | NM_001003899.2 | Neuropathology | NM_001003899.2:3060 | 10.17295873 | 10.18577365 | 0.012814916 |
| Cluster1 | Nos2 | NM_010927.3 | Neuropathology | NM_010927.3:3715 | 3.793895883 | 3.807870078 | 0.013974196 |
| Cluster1 | AI464131 | NM_001085515.2 | Neuropathology | NM_001085515.2:1232 | 6.408542042 | 6.42483813 | 0.016296088 |
| Cluster1 | Egfl7 | NM_001164564.1 | Neuropathology | NM_001164564.1:168 | 7.850343134 | 7.870518845 | 0.020175711 |
| Cluster1 | Shank2 | NM_001081370.2 | Neuropathology | NM_001081370.2:4930 | 7.875565068 | 7.897905945 | 0.022340877 |
| Cluster1 | Col4a2 | NM_009932.3 | Neuropathology | NM_009932.3:5600 | 7.45971849 | 7.484339566 | 0.024621076 |
| Cluster1 | Notch3 | NM_008716.2 | Neuropathology | NM_008716.2:550 | 7.12763328 | 7.153906645 | 0.026273366 |
| Cluster1 | Cadm3 | NM_053199.3 | Neuropathology | NM_053199.3:3295 | 7.936932337 | 7.97053734 | 0.033605004 |
| Cluster1 | Taf4 | NM_001081092.1 | Neuropathology | NM_001081092.1:3016 | 8.646000604 | 8.679831933 | 0.033831329 |
| Cluster1 | Pllp | NM_026385.3 | Neuropathology | NM_026385.3:345 | 7.077189581 | 7.11701966 | 0.039830079 |
| Cluster1 | Nmb | NM_026523.2 | Neuropathology | NM_026523.2:500 | 7.231749192 | 7.273282757 | 0.041533565 |
| Cluster1 | Esam | NM_027102.3 | Neuropathology | NM_027102.3:495 | 7.042042359 | 7.084648965 | 0.042606606 |
| Cluster1 | Olig2 | NM_016967.2 | Neuropathology | NM_016967.2:1740 | 8.957348759 | 9.001506717 | 0.044157957 |
| Cluster1 | Bad | NM_007522.3 | Neuropathology | NM_007522.3:1146 | 8.60659028 | 8.653973597 | 0.047383317 |
| Cluster1 | Hras | NM_001130443.1 | Neuropathology | NM_001130443.1:240 | 9.281756198 | 9.334217409 | 0.052461211 |
| Cluster1 | Irf8 | NM_008320.3 | Neuropathology | NM_008320.3:2274 | 5.513490746 | 5.570918255 | 0.05742751 |
| Cluster1 | Clu | NM_013492.2 | Neuropathology | NM_013492.2:354 | 12.51028204 | 12.57297136 | 0.062689322 |
| Cluster1 | Dnah1 | NM_001033668.1 | Neuropathology | NM_001033668.1:6114 | 6.249350524 | 6.312338439 | 0.062987916 |
| Cluster1 | Pmp22 | NM_008885.2 | Neuropathology | NM_008885.2:395 | 6.646018611 | 6.710530952 | 0.064512342 |
| Cluster1 | Ccnd1 | NM_007631.1 | Neuropathology | NM_007631.1:2000 | 9.596133837 | 9.666135219 | 0.070001382 |
| Cluster1 | Grn | NM_008175.3 | Neuropathology | NM_008175.3:2010 | 10.07767027 | 10.15241161 | 0.074741337 |
| Cluster1 | Rras | NM_009101.2 | Neuropathology | NM_009101.2:282 | 6.394462695 | 6.47037417 | 0.075911476 |
| Cluster1 | Mmp9 | NM_013599.2 | Neuropathology | NM_013599.2:1570 | 8.920144401 | 8.998463501 | 0.078319099 |
| Cluster1 | Erg | NM_133659.2 | Neuropathology | NM_133659.2:992 | 4.448900951 | 4.530445355 | 0.081544404 |
| Cluster1 | Plekho2 | NM_153119.2 | Neuropathology | NM_153119.2:406 | 7.08952993 | 7.171777274 | 0.082247344 |
| Cluster1 | Plcb3 | NM_008874.3 | Neuropathology | NM_008874.3:1880 | 8.019840885 | 8.107033948 | 0.087193063 |
| Cluster1 | Cln3 | NM_001146311.1 | Neuropathology | NM_001146311.1:378 | 7.766363262 | 7.853715013 | 0.08735175 |
| Cluster1 | Drd1 | NM_010076.3 | Neuropathology | NM_010076.3:1785 | 3.432959407 | 3.521678952 | 0.088719544 |
| Cluster1 | Gnai2 | NM_008138.4 | Neuropathology | NM_008138.4:971 | 10.93111422 | 11.02322416 | 0.092109937 |
| Cluster1 | Cx3cr1 | NM_009987.3 | Neuropathology | NM_009987.3:2696 | 7.064796646 | 7.157700618 | 0.092903972 |
| Cluster1 | Adcy9 | NM_009624.1 | Neuropathology | NM_009624.1:3640 | 7.101923161 | 7.1959381 | 0.09401494 |
| Cluster1 | Kcnj10 | NM_001039484.1 | Neuropathology | NM_001039484.1:400 | 6.958842675 | 7.054577187 | 0.095734511 |
| Cluster1 | Ring1 | NM_009066.3 | Neuropathology | NM_009066.3:243 | 8.519223434 | 8.615004242 | 0.095780808 |
| Cluster1 | Arc | NM_018790.2 | Neuropathology | NM_018790.2:2715 | 3.802193217 | 3.910252963 | 0.108059746 |
| Cluster1 | Actn1 | NM_134156.2 | Neuropathology | NM_134156.2:2688 | 10.05477388 | 10.16373143 | 0.108957553 |
| Cluster1 | Apoe | NM_001305844.1 | Neuropathology | NM_001305844.1:903 | 10.93577648 | 11.04550031 | 0.109723827 |
| Cluster1 | Col4a1 | NM_009931.2 | Neuropathology | NM_009931.2:4116 | 7.746514321 | 7.861552216 | 0.115037895 |
| Cluster1 | Camk2g | NM_001039138.1 | Neuropathology | NM_001039138.1:2525 | 8.599875659 | 8.715206994 | 0.115331335 |
| Cluster1 | Trem2 | NM_031254.2 | Neuropathology | NM_031254.2:646 | 4.844737144 | 4.960928667 | 0.116191523 |
| Cluster1 | Aif1 | NM_019467.2 | Neuropathology | NM_019467.2:55 | 3.203984166 | 3.321206567 | 0.117222401 |
| Cluster1 | Lamb2 | NM_008483.3 | Neuropathology | NM_008483.3:712 | 8.175225173 | 8.294000349 | 0.118775176 |
| Cluster1 | Pfn1 | NM_011072.4 | Neuropathology | NM_011072.4:266 | 11.21419464 | 11.34103506 | 0.126840424 |
| Cluster1 | Tor1a | NM_144884.1 | Neuropathology | NM_144884.1:220 | 8.339649615 | 8.47112648 | 0.131476865 |
| Cluster1 | Pla2g4f | NM_001024145.2 | Neuropathology | NM_001024145.2:2002 | 3.529196268 | 3.661635602 | 0.132439334 |
| Cluster1 | Flt4 | NM_008029.3 | Neuropathology | NM_008029.3:5690 | 6.952042295 | 7.086667018 | 0.134624723 |
| Cluster1 | Stat3 | NM_213659.2 | Neuropathology | NM_213659.2:1360 | 9.358222614 | 9.502125695 | 0.143903081 |
| Cluster1 | Cacna1d | NM_028981.2 | Neuropathology | NM_028981.2:2935 | 9.714297042 | 9.859154262 | 0.144857221 |
| Cluster1 | Snrpa | NM_001046637.1 | Neuropathology | NM_001046637.1:880 | 8.557884865 | 8.704249502 | 0.146364637 |
| Cluster1 | Akt1s1 | NM_026270.4 | Neuropathology | NM_026270.4:946 | 8.454443247 | 8.601046474 | 0.146603227 |
| Cluster1 | Adcyap1 | NM_001315503.1 | Neuropathology | NM_001315503.1:2625 | 3.454175893 | 3.601696516 | 0.147520623 |
| Cluster1 | Tspo | NM_009775.4 | Neuropathology | NM_009775.4:241 | 7.255453524 | 7.409051439 | 0.153597915 |
| Cluster1 | Arsa | NM_009713.4 | Neuropathology | NM_009713.4:2802 | 8.37332195 | 8.527535651 | 0.154213701 |
| Cluster1 | Mapkapk2 | NM_008551.1 | Neuropathology | NM_008551.1:1991 | 7.09386648 | 7.249587556 | 0.155721076 |
| Cluster1 | Ddx23 | NM_001080981.1 | Neuropathology | NM_001080981.1:2175 | 9.795081822 | 9.954727043 | 0.159645221 |
| Cluster1 | Dagla | NM_198114.2 | Neuropathology | NM_198114.2:685 | 7.251624424 | 7.425593578 | 0.173969153 |
| Cluster1 | Acin1 | NM_001085472.2 | Neuropathology | NM_001085472.2:674 | 10.78641258 | 10.96472351 | 0.178310931 |
| Cluster1 | Ap1s1 | NM_007457.2 | Neuropathology | NM_007457.2:848 | 8.840762191 | 9.022811651 | 0.18204946 |
| Cluster1 | Atp6v0e2 | NM_133764.3 | Neuropathology | NM_133764.3:882 | 10.0362904 | 10.21913221 | 0.182841813 |
| Cluster1 | Phf2 | NM_011078.2 | Neuropathology | NM_011078.2:3590 | 8.708307961 | 8.891510325 | 0.183202363 |
| Cluster1 | Pla2g4b | XM_925095.2 | Neuropathology | XM_925095.2:310 | 5.729824644 | 5.917073663 | 0.187249019 |
| Cluster1 | Csnk2a2 | NM_009974.3 | Neuropathology | NM_009974.3:1066 | 8.797969484 | 8.98688019 | 0.188910706 |
| Cluster1 | Gata2 | NM_008090.4 | Neuropathology | NM_008090.4:2960 | 8.346668853 | 8.538091908 | 0.191423055 |
| Cluster1 | Sh3tc2 | NM_172628.2 | Neuropathology | NM_172628.2:2150 | 4.718361626 | 4.912410354 | 0.194048728 |
| Cluster1 | Mfn2 | XM_006535920.1 | Neuropathology | XM_006535920.1:692 | 9.31596467 | 9.510348802 | 0.194384132 |
| Cluster1 | Xab2 | NM_026156.2 | Neuropathology | NM_026156.2:1616 | 9.039385184 | 9.234194723 | 0.194809539 |
| Cluster1 | Vegfa | NM_001025250.3 | Neuropathology | NM_001025250.3:3015 | 7.462420473 | 7.658532839 | 0.196112366 |

| | | | | | | | |
|---|---|---|---|---|---|---|---|
| Cluster1 | Usp21 | NM_013919.4 | Neuropathology | NM_013919.4:1590 | 8.136811725 | 8.33650656 | 0.199694835 |
| Cluster1 | Atp8a2 | NM_015803.2 | Neuropathology | NM_015803.2:185 | 8.77310623 | 8.975117123 | 0.202010893 |
| Cluster1 | Sptbn2 | NM_021287.1 | Neuropathology | NM_021287.1:6145 | 6.800123353 | 7.004051885 | 0.203928532 |
| Cluster1 | Crebbp | NM_001025432.1 | Neuropathology | NM_001025432.1:3770 | 10.57630955 | 10.78400087 | 0.207691319 |
| Cluster1 | Adora2a | NM_009630.2 | Neuropathology | NM_009630.2:2306 | 2.165107985 | 2.372952098 | 0.207844113 |
| Cluster1 | Atp6v0c | NM_009729.3 | Neuropathology | NM_009729.3:569 | 12.26069759 | 12.46895999 | 0.208262402 |
| Cluster1 | Cacnb2 | NM_023116.3 | Neuropathology | NM_023116.3:735 | 6.800188081 | 7.018144529 | 0.217956448 |
| Cluster1 | Lsm7 | NM_025349.2 | Neuropathology | NM_025349.2:37 | 9.029149163 | 9.251115477 | 0.221966313 |
| Cluster1 | Sox9 | NM_011448.4 | Neuropathology | NM_011448.4:3540 | 8.58500007 | 8.808175865 | 0.223175795 |
| Cluster1 | Ap3m2 | NM_029505.3 | Neuropathology | NM_029505.3:1646 | 8.803662957 | 9.032500373 | 0.228837416 |
| Cluster1 | Cdk5 | NM_007668.3 | Neuropathology | NM_007668.3:77 | 8.450097746 | 8.680658406 | 0.230560659 |
| Cluster1 | Sncb | NM_033610.2 | Neuropathology | NM_033610.2:676 | 3.110196178 | 3.341274184 | 0.231078006 |
| Cluster1 | Keap1 | NM_016679.4 | Neuropathology | NM_016679.4:4140 | 7.914444932 | 8.147077698 | 0.232632766 |
| Cluster1 | Psmb9 | NM_013585.2 | Neuropathology | NM_013585.2:540 | 5.575312331 | 5.810571635 | 0.235259304 |
| Cluster1 | Sqstm1 | NM_011018.2 | Neuropathology | NM_011018.2:1430 | 10.68769061 | 10.92523916 | 0.237548554 |
| Cluster1 | Psen2 | NM_001128605.1 | Neuropathology | NM_001128605.1:560 | 9.890317484 | 10.12890941 | 0.23859193 |
| Cluster1 | Atp6v1e1 | NM_007510.2 | Neuropathology | NM_007510.2:1025 | 11.15882109 | 11.39770404 | 0.238882951 |
| Cluster1 | Ltbr | NM_010736.3 | Neuropathology | NM_010736.3:1962 | 8.423557162 | 8.663842702 | 0.240285539 |
| Cluster1 | Cldn5 | NM_013805.4 | Neuropathology | NM_013805.4:975 | 9.055309554 | 9.296790052 | 0.241480498 |
| Cluster1 | Prkcsh | NM_008925.1 | Neuropathology | NM_008925.1:1295 | 10.54164357 | 10.78358761 | 0.241944038 |
| Cluster1 | Stx2 | NM_007941.2 | Neuropathology | NM_007941.2:225 | 10.42850674 | 10.67131549 | 0.242808752 |
| Cluster1 | Adra2a | NM_007417.4 | Neuropathology | NM_007417.4:3594 | 7.560523907 | 7.805163453 | 0.244639547 |
| Cluster1 | Gtf2ird1 | NM_001081464.1 | Neuropathology | NM_001081464.1:1430 | 9.846187708 | 10.09724866 | 0.251060948 |
| Cluster1 | Casp7 | NM_007611.2 | Neuropathology | NM_007611.2:1468 | 7.869377924 | 8.131754091 | 0.262376167 |
| Cluster1 | C4a | NM_011413.2 | Neuropathology | NM_011413.2:4186 | 4.304511042 | 4.568032105 | 0.263521063 |
| Cluster1 | Rac1 | NM_009007.2 | Neuropathology | NM_009007.2:1045 | 10.69945099 | 10.96951963 | 0.270068637 |
| Cluster1 | Park7 | NM_020569.3 | Neuropathology | NM_020569.3:334 | 10.86000448 | 11.13208196 | 0.272077484 |
| Cluster1 | Ctns | NM_031251.4 | Neuropathology | NM_031251.4:1854 | 7.415572775 | 7.692441147 | 0.276868372 |
| Cluster1 | Cacna1b | NM_001042528.1 | Neuropathology | NM_001042528.1:4345 | 8.383380298 | 8.672425342 | 0.289045044 |
| Cluster1 | Bax | NM_007527.3 | Neuropathology | NM_007527.3:735 | 7.472690839 | 7.7756432 | 0.302952361 |
| Cluster1 | Wfs1 | NM_011716.2 | Neuropathology | NM_011716.2:2975 | 7.070120944 | 7.374995976 | 0.304875032 |
| Cluster1 | Myrf | NM_001033481.1 | Neuropathology | NM_001033481.1:4465 | 4.068240861 | 4.373648211 | 0.30540735 |
| Cluster1 | Lsr | NM_001164184.1 | Neuropathology | NM_001164184.1:445 | 9.40842528 | 9.716836606 | 0.308411325 |
| Cluster1 | Cdk5rap3 | NM_030248.1 | Neuropathology | NM_030248.1:248 | 8.317570895 | 8.626676403 | 0.309105508 |
| Cluster1 | Cd9 | NM_007657.3 | Neuropathology | NM_007657.3:620 | 11.90856338 | 12.22209602 | 0.313532645 |
| Cluster1 | Grin2c | NM_010350.2 | Neuropathology | NM_010350.2:2408 | 3.137503524 | 3.462052319 | 0.324548795 |
| Cluster1 | Tnf | NM_013693.2 | Neuropathology | NM_013693.2:514 | 2.852997588 | 3.17951105 | 0.326513463 |
| Cluster1 | Prf1 | NM_011073.2 | Neuropathology | NM_011073.2:1350 | 2.947666157 | 3.279471296 | 0.331805139 |
| Cluster1 | Cd8a | NM_001081110.2 | Neuropathology | NM_001081110.2:355 | 3.152183419 | 3.491853096 | 0.339669677 |
| Cluster1 | Grin2d | NM_008172.2 | Neuropathology | NM_008172.2:1201 | 4.486392594 | 4.829342194 | 0.3429496 |
| Cluster1 | Tie1 | NM_011587.2 | Neuropathology | NM_011587.2:2715 | 6.609695873 | 6.953847229 | 0.344151356 |
| Cluster1 | Tenm2 | NM_011856.3 | Neuropathology | NM_011856.3:1452 | 10.28220811 | 10.63369911 | 0.351491001 |
| Cluster1 | Nsf | NM_008740.2 | Neuropathology | NM_008740.2:395 | 10.30320995 | 10.66000481 | 0.35679486 |
| Cluster1 | Slc1a2 | NM_001077514.3 | Neuropathology | NM_001077514.3:1675 | 9.907671844 | 10.26988641 | 0.362214564 |
| Cluster1 | Polr2l | NM_025593.1 | Neuropathology | NM_025593.1:348 | 6.936461272 | 7.303872055 | 0.367410783 |
| Cluster1 | Aldh1l1 | NM_027406.1 | Neuropathology | NM_027406.1:1340 | 8.111370448 | 8.48458125 | 0.373210803 |
| Cluster1 | Efna1 | NM_010107.4 | Neuropathology | NM_010107.4:437 | 7.560332834 | 7.950585057 | 0.390252223 |
| Cluster1 | Il6ra | NM_010559.2 | Neuropathology | NM_010559.2:2825 | 5.646018611 | 6.04187814 | 0.395859529 |
| Cluster1 | Kcnb1 | NM_008420.3 | Neuropathology | NM_008420.3:3555 | 7.718327374 | 8.119485873 | 0.401158499 |
| Cluster1 | Ncf1 | NM_001286037.1 | Neuropathology | NM_001286037.1:970 | 6.086295483 | 6.492894667 | 0.406599183 |
| Cluster1 | Ngf | NM_001112698.1 | Neuropathology | NM_001112698.1:630 | 4.704595348 | 5.112700133 | 0.408104785 |
| Cluster1 | Adcy5 | NM_001012765.4 | Neuropathology | NM_001012765.4:219 | 2.600507645 | 3.030336078 | 0.429828433 |
| Cluster1 | Mmrn2 | NM_153127.3 | Neuropathology | NM_153127.3:2622 | 5.983677695 | 6.438875003 | 0.455197308 |
| Cluster1 | Htr1a | NM_008308.4 | Neuropathology | NM_008308.4:3170 | 2.869871406 | 3.354028938 | 0.484157532 |
| Cluster1 | Slc11a1 | NM_013612.2 | Neuropathology | NM_013612.2:945 | 3.658782734 | 4.149340669 | 0.490557935 |
| Cluster1 | Ggt1 | NM_008116.2 | Neuropathology | NM_008116.2:1025 | 8.583289658 | 9.079964713 | 0.496675055 |
| Cluster1 | Acvrl1 | NM_009612.2 | Neuropathology | NM_009612.2:2985 | 5.10706011 | 5.626585151 | 0.519525041 |
| Cluster1 | Shh | NM_009170.3 | Neuropathology | NM_009170.3:2055 | 3.096767855 | 3.617651119 | 0.520883265 |
| Cluster1 | Cxcl12 | NM_021704.3 | Neuropathology | NM_021704.3:259 | 11.00695366 | 11.54828248 | 0.541328824 |
| Cluster1 | Inhbb | NM_008381.3 | Neuropathology | NM_008381.3:1084 | 3.363871925 | 3.963011648 | 0.599139723 |
| Cluster1 | Sox10 | XM_128139.6 | Neuropathology | XM_128139.6:2646 | 6.006073567 | 6.632559052 | 0.626485485 |
| Cluster1 | Epo | NM_007942.2 | Neuropathology | NM_007942.2:216 | 2.340562269 | 3.193771743 | 0.853209474 |
| Cluster1 | F2 | NM_010168.2 | Neuropathology | NM_010168.2:1010 | 3.280213996 | 4.145269857 | 0.865055861 |
| Cluster1 | Klk6 | NM_001164696.1 | Neuropathology | NM_001164696.1:1053 | 3.08236197 | 4.205157884 | 1.122795914 |
| Cluster2 | Mmp14 | NM_008608.3 | Neuropathology | NM_008608.3:554 | 9.993943201 | 9.713197466 | -0.280745735 |
| Cluster2 | Axin2 | NM_015732.4 | Neuropathology | NM_015732.4:1120 | 7.663913842 | 7.571221725 | -0.092692117 |
| Cluster2 | Amigo1 | NM_001004293.2 | Neuropathology | NM_001004293.2:925 | 8.44412453 | 8.357442039 | -0.086682491 |
| Cluster2 | Hdac7 | NM_019572.2 | Neuropathology | NM_019572.2:3706 | 7.838384558 | 7.7601544 | -0.078230158 |
| Cluster2 | Dot1l | NM_199322.1 | Neuropathology | NM_199322.1:5490 | 6.745439037 | 6.667537367 | -0.07790167 |
| Cluster2 | Cdk5r1 | NM_009871.2 | Neuropathology | NM_009871.2:3280 | 6.982765463 | 6.921424429 | -0.061341034 |
| Cluster2 | Comt | NM_007744.3 | Neuropathology | NM_007744.3:625 | 8.170025185 | 8.170175448 | 0.000150262 |
| Cluster2 | Src | NM_001025395.2 | Neuropathology | NM_001025395.2:968 | 8.838951767 | 8.852264198 | 0.013312431 |
| Cluster2 | Tnfrsf1a | NM_011609.2 | Neuropathology | NM_011609.2:615 | 8.510645504 | 8.530464863 | 0.019819359 |
| Cluster2 | Cers2 | NM_029789.1 | Neuropathology | NM_029789.1:1422 | 8.989380307 | 9.013308714 | 0.023928407 |
| Cluster2 | Il4ra | NM_001008700.3 | Neuropathology | NM_001008700.3:2908 | 6.829025 | 6.868760834 | 0.039735834 |
| Cluster2 | Gaa | NM_008064.3 | Neuropathology | NM_008064.3:1390 | 9.159076627 | 9.205292311 | 0.046215685 |
| Cluster2 | Tgfb1 | NM_011577.1 | Neuropathology | NM_011577.1:1470 | 8.284569655 | 8.35985935 | 0.075289696 |
| Cluster2 | Fos | NM_010234.2 | Neuropathology | NM_010234.2:1330 | 6.822347758 | 6.90346012 | 0.081112362 |
| Cluster2 | Bace1 | NM_011792.4 | Neuropathology | NM_011792.4:3107 | 10.07337843 | 10.16324083 | 0.089862408 |
| Cluster2 | Mthfr | NR_027809.1 | Neuropathology | NR_027809.1:862 | 4.979796718 | 5.077242999 | 0.097446281 |

| | | | | | | | |
|---|---|---|---|---|---|---|---|
| Cluster2 | Naglu | NM_013792.2 | Neuropathology | NM_013792.2:2334 | 8.652611972 | 8.788522589 | 0.135910617 |
| Cluster2 | Mapk3 | NM_011952.2 | Neuropathology | NM_011952.2:825 | 9.200604398 | 9.353621028 | 0.15301663 |
| Cluster2 | Prkaca | NM_008854.3 | Neuropathology | NM_008854.3:699 | 10.32239577 | 10.47925987 | 0.156864103 |
| Cluster2 | Rela | NM_009045.4 | Neuropathology | NM_009045.4:645 | 7.621319301 | 7.781785901 | 0.1604666 |
| Cluster2 | Usp30 | NM_001033202.3 | Neuropathology | NM_001033202.3:2576 | 8.06010144 | 8.222311912 | 0.162210471 |
| Cluster2 | Mbp | NM_010777.3 | Neuropathology | NM_010777.3:761 | 5.518535139 | 5.686640588 | 0.168105449 |
| Cluster2 | Sf3a2 | NM_013651.4 | Neuropathology | NM_013651.4:494 | 9.300524163 | 9.475409193 | 0.17488503 |
| Cluster2 | Mta1 | NM_054081.2 | Neuropathology | NM_054081.2:955 | 10.37748711 | 10.55406597 | 0.176578861 |
| Cluster2 | Atp13a2 | NM_029097.2 | Neuropathology | NM_029097.2:1360 | 8.080737598 | 8.263457008 | 0.182719411 |
| Cluster2 | Stx1a | NM_016801.3 | Neuropathology | NM_016801.3:72 | 9.65115038 | 9.847417583 | 0.196267204 |
| Cluster2 | Entpd2 | NM_009849.2 | Neuropathology | NM_009849.2:1016 | 6.469397617 | 6.668743189 | 0.199345572 |
| Cluster2 | Cxxc1 | NM_028868.3 | Neuropathology | NM_028868.3:1749 | 8.717556479 | 8.9202933 | 0.202736821 |
| Cluster2 | Acaa1a | NM_130864.3 | Neuropathology | NM_130864.3:626 | 8.747672715 | 8.957522887 | 0.209850173 |
| Cluster2 | Syt7 | NM_018801.3 | Neuropathology | NM_018801.3:990 | 10.12743983 | 10.36179029 | 0.23435046 |
| Cluster2 | Htra2 | NM_019752.3 | Neuropathology | NM_019752.3:1088 | 7.029342449 | 7.272396509 | 0.24305406 |
| Cluster2 | Palm | NM_023128.4 | Neuropathology | NM_023128.4:548 | 9.810250286 | 10.07524514 | 0.264994858 |
| Cluster2 | Map2k2 | NM_023138.4 | Neuropathology | NM_023138.4:1440 | 9.603088479 | 9.87175126 | 0.268662781 |
| Cluster2 | Mapt | NM_001038609.2 | Neuropathology | NM_001038609.2:1202 | 7.41502341 | 7.710875296 | 0.295851885 |
| Cluster2 | Fam104a | NM_138598.5 | Neuropathology | NM_138598.5:1120 | 8.524149891 | 8.831418094 | 0.307268203 |
| Cluster3 | Phf21a | NM_001109690.1 | Neuropathology | NM_001109690.1:2324 | 6.080870812 | 5.970623614 | -0.110247197 |
| Cluster3 | Axin2 | NM_015732.4 | Neuropathology | NM_015732.4:1120 | 7.663913842 | 7.571221725 | -0.092692117 |
| Cluster3 | Fyn | NM_008054.2 | Neuropathology | NM_008054.2:1030 | 8.634339148 | 8.543650538 | -0.09068861 |
| Cluster3 | Hdac7 | NM_019572.2 | Neuropathology | NM_019572.2:3706 | 7.838384558 | 7.7601544 | -0.078230158 |
| Cluster3 | Dot1l | NM_199322.1 | Neuropathology | NM_199322.1:5490 | 6.745439037 | 6.667537367 | -0.07790167 |
| Cluster3 | Cdk5r1 | NM_009871.2 | Neuropathology | NM_009871.2:3280 | 6.982765463 | 6.921424429 | -0.061341034 |
| Cluster3 | Abl1 | NM_009594.4 | Neuropathology | NM_009594.4:1378 | 7.615887074 | 7.615887074 | 0 |
| Cluster3 | Adam10 | NM_007399.3 | Neuropathology | NM_007399.3:2390 | 9.610175903 | 9.620806317 | 0.010630413 |
| Cluster3 | Src | NM_001025395.2 | Neuropathology | NM_001025395.2:968 | 8.838951767 | 8.852264198 | 0.013312431 |
| Cluster3 | Cers2 | NM_029789.1 | Neuropathology | NM_029789.1:1422 | 8.989380307 | 9.013308714 | 0.023928407 |
| Cluster3 | Ran | NM_009391.3 | Neuropathology | NM_009391.3:1755 | 8.308634821 | 8.350232021 | 0.041597201 |
| Cluster3 | Akt2 | NM_001110208.1 | Neuropathology | NM_001110208.1:2504 | 7.895393514 | 7.964976149 | 0.069582635 |
| Cluster3 | Htt | NM_010414.1 | Neuropathology | NM_010414.1:2300 | 8.591541046 | 8.668477286 | 0.076936239 |
| Cluster3 | Efr3a | NM_133766.3 | Neuropathology | NM_133766.3:1062 | 9.230789028 | 9.316168375 | 0.085379347 |
| Cluster3 | Bace1 | NM_011792.4 | Neuropathology | NM_011792.4:3107 | 10.07337843 | 10.16324083 | 0.089862408 |
| Cluster3 | Sp1 | NM_013672.2 | Neuropathology | NM_013672.2:6580 | 9.100057805 | 9.19128134 | 0.091223536 |
| Cluster3 | Mthfr | NR_027809.1 | Neuropathology | NR_027809.1:862 | 4.979796718 | 5.077242999 | 0.097446281 |
| Cluster3 | Camk2g | NM_001039138.1 | Neuropathology | NM_001039138.1:2525 | 8.599875659 | 8.715206994 | 0.115331335 |
| Cluster3 | Creb1 | NM_001037726.1 | Neuropathology | NM_001037726.1:2734 | 8.891905188 | 9.011115414 | 0.119210226 |
| Cluster3 | Tcerg1 | NM_001039474.1 | Neuropathology | NM_001039474.1:468 | 9.036008724 | 9.172277481 | 0.136268757 |
| Cluster3 | Atxn2 | NM_009125.2 | Neuropathology | NM_009125.2:3036 | 9.467361005 | 9.61225112 | 0.144890115 |
| Cluster3 | Myh10 | NM_175260.2 | Neuropathology | NM_175260.2:2540 | 9.734235417 | 9.884254483 | 0.150019066 |
| Cluster3 | Ep300 | NM_177821.6 | Neuropathology | NM_177821.6:4305 | 9.995152356 | 10.14986776 | 0.154715407 |
| Cluster3 | Trim37 | NM_197987.2 | Neuropathology | NM_197987.2:972 | 9.688547561 | 9.8454038 | 0.156856239 |
| Cluster3 | Prkaca | NM_008854.3 | Neuropathology | NM_008854.3:699 | 10.32239577 | 10.47925987 | 0.156864103 |
| Cluster3 | Meaf6 | NM_027310.3 | Neuropathology | NM_027310.3:178 | 8.50670428 | 8.667147032 | 0.160442752 |
| Cluster3 | Rela | NM_009045.4 | Neuropathology | NM_009045.4:645 | 7.621319301 | 7.781785901 | 0.1604666 |
| Cluster3 | Ube2k | NM_016786.3 | Neuropathology | NM_016786.3:1212 | 10.13594651 | 10.29728314 | 0.161336632 |
| Cluster3 | Usp30 | NM_001033202.3 | Neuropathology | NM_001033202.3:2576 | 8.06010144 | 8.222311912 | 0.162210471 |
| Cluster3 | Mbp | NM_010777.3 | Neuropathology | NM_010777.3:761 | 5.518535139 | 5.686640588 | 0.168105449 |
| Cluster3 | Sf3a2 | NM_013651.4 | Neuropathology | NM_013651.4:494 | 9.300524163 | 9.475409193 | 0.17488503 |
| Cluster3 | Mta1 | NM_054081.2 | Neuropathology | NM_054081.2:955 | 10.37748711 | 10.55406597 | 0.176578861 |
| Cluster3 | Gnptab | NM_001004164.2 | Neuropathology | NM_001004164.2:1214 | 8.653239196 | 8.836318665 | 0.183079469 |
| Cluster3 | Mapk9 | NM_207692.1 | Neuropathology | NM_207692.1:260 | 9.037107627 | 9.225111013 | 0.188003386 |
| Cluster3 | Ppp2r5c | NM_001135001.1 | Neuropathology | NM_001135001.1:1400 | 10.67437326 | 10.86401973 | 0.189646474 |
| Cluster3 | Calm1 | NM_009790.4 | Neuropathology | NM_009790.4:1575 | 13.16364889 | 13.35365548 | 0.190006594 |
| Cluster3 | Hdac2 | NM_008229.2 | Neuropathology | NM_008229.2:1010 | 10.52948424 | 10.72132139 | 0.191837148 |
| Cluster3 | Ppp2ca | NM_019411.4 | Neuropathology | NM_019411.4:975 | 10.95846572 | 11.15101019 | 0.192544478 |
| Cluster3 | Ppm1l | NM_178726.3 | Neuropathology | NM_178726.3:2320 | 7.994042234 | 8.189453956 | 0.195411722 |
| Cluster3 | Hif1a | NM_010431.2 | Neuropathology | NM_010431.2:1294 | 10.84845081 | 11.04554126 | 0.197090453 |
| Cluster3 | Fus | NM_139149.2 | Neuropathology | NM_139149.2:15 | 10.75637285 | 10.95358173 | 0.197208878 |
| Cluster3 | Atxn7 | NM_139227.4 | Neuropathology | NM_139227.4:3328 | 8.710978583 | 8.908452669 | 0.197474087 |
| Cluster3 | Mto1 | NM_026658.2 | Neuropathology | NM_026658.2:1058 | 8.071784117 | 8.277171143 | 0.205387025 |
| Cluster3 | Ap3s1 | NM_009681.5 | Neuropathology | NM_009681.5:610 | 9.93799167 | 10.1436957 | 0.20570403 |
| Cluster3 | Pten | NM_008960.2 | Neuropathology | NM_008960.2:5160 | 11.19622096 | 11.40545263 | 0.209231667 |
| Cluster3 | Acaa1a | NM_130864.3 | Neuropathology | NM_130864.3:626 | 8.747672715 | 8.957522887 | 0.209850173 |
| Cluster3 | Mapk1 | NM_011949.3 | Neuropathology | NM_011949.3:1210 | 11.02964613 | 11.24065123 | 0.211005099 |
| Cluster3 | Ube3a | NM_173010.3 | Neuropathology | NM_173010.3:2025 | 10.48277267 | 10.6945886 | 0.211815925 |
| Cluster3 | Cul1 | NM_012042.3 | Neuropathology | NM_012042.3:919 | 10.19884403 | 10.41496001 | 0.216115979 |
| Cluster3 | Psen1 | NM_008943.2 | Neuropathology | NM_008943.2:2770 | 9.177232648 | 9.39693652 | 0.219703872 |
| Cluster3 | Fmr1 | NM_008031.2 | Neuropathology | NM_008031.2:765 | 10.17819176 | 10.40036181 | 0.22217005 |
| Cluster3 | Gtf2h1 | NM_008186.4 | Neuropathology | NM_008186.4:830 | 7.890659482 | 8.123811021 | 0.23315154 |
| Cluster3 | Syt7 | NM_018801.3 | Neuropathology | NM_018801.3:990 | 10.12743983 | 10.36179029 | 0.23435046 |
| Cluster3 | Rad23b | NM_009011.4 | Neuropathology | NM_009011.4:1585 | 10.48840756 | 10.72497848 | 0.23657092 |
| Cluster3 | Lamp1 | NM_010684.2 | Neuropathology | NM_010684.2:2080 | 11.75582854 | 11.99306221 | 0.237233676 |
| Cluster3 | Pdpk1 | NM_001080773.2 | Neuropathology | NM_001080773.2:856 | 9.537247526 | 9.775651429 | 0.238403904 |
| Cluster3 | Cul2 | NM_029402.3 | Neuropathology | NM_029402.3:2528 | 8.652647821 | 8.892937398 | 0.240289577 |
| Cluster3 | Htra2 | NM_019752.3 | Neuropathology | NM_019752.3:1088 | 7.029342449 | 7.272396509 | 0.24305406 |
| Cluster3 | Plcl2 | NM_013880.3 | Neuropathology | NM_013880.3:475 | 8.904453991 | 9.149188221 | 0.24473423 |
| Cluster3 | P2rx4 | NM_011026.2 | Neuropathology | NM_011026.2:1655 | 8.898359532 | 9.147790026 | 0.249430494 |
| Cluster3 | Trim28 | NM_011588.3 | Neuropathology | NM_011588.3:1615 | 9.751619372 | 10.00136597 | 0.249746597 |

| | | | | | | | |
|---|---|---|---|---|---|---|---|
| Cluster3 | Rab3a | NM_009001.6 | Neuropathology | NM_009001.6:1272 | 10.45894996 | 10.71003581 | 0.251085858 |
| Cluster3 | Atf4 | NM_009716.2 | Neuropathology | NM_009716.2:812 | 11.41020752 | 11.6639383 | 0.253730771 |
| Cluster3 | Lypla1 | XM_006495472.2 | Neuropathology | XM_006495472.2:648 | 9.400794067 | 9.655673823 | 0.254879756 |
| Cluster3 | Rhoa | NM_016802.4 | Neuropathology | NM_016802.4:1885 | 10.89815544 | 11.15541911 | 0.257263671 |
| Cluster3 | Palm | NM_023128.4 | Neuropathology | NM_023128.4:548 | 9.810250286 | 10.07524514 | 0.264994858 |
| Cluster3 | Rab2a | NM_021518.3 | Neuropathology | NM_021518.3:450 | 12.41470637 | 12.67991548 | 0.265209107 |
| Cluster3 | Srsf4 | NM_020587.2 | Neuropathology | NM_020587.2:524 | 9.059114585 | 9.32690132 | 0.267786735 |
| Cluster3 | Map2k2 | NM_023138.4 | Neuropathology | NM_023138.4:1440 | 9.603088479 | 9.87175126 | 0.268662781 |
| Cluster3 | Atp6v1h | XM_006495434.2 | Neuropathology | XM_006495434.2:1296 | 11.18905545 | 11.45925742 | 0.270201967 |
| Cluster3 | Sod1 | NM_011434.1 | Neuropathology | NM_011434.1:406 | 13.26643007 | 13.54061544 | 0.274185367 |
| Cluster3 | Spast | NM_016962.2 | Neuropathology | NM_016962.2:1530 | 9.989706707 | 10.26474753 | 0.275040821 |
| Cluster3 | Ugcg | NM_011673.3 | Neuropathology | NM_011673.3:610 | 9.539478728 | 9.826469042 | 0.286990313 |
| Cluster3 | Atrn | NM_009730.2 | Neuropathology | NM_009730.2:1375 | 9.636760546 | 9.92662957 | 0.289869024 |
| Cluster3 | Mapt | NM_001038609.2 | Neuropathology | NM_001038609.2:1202 | 7.41502341 | 7.710875296 | 0.295851885 |
| Cluster3 | Cab39 | NM_133781.4 | Neuropathology | NM_133781.4:2830 | 10.4487616 | 10.74807122 | 0.299309617 |
| Cluster3 | Sucla2 | NM_011506.1 | Neuropathology | NM_011506.1:955 | 10.66181817 | 10.96385339 | 0.302035216 |
| Cluster3 | Mta2 | NM_011842.3 | Neuropathology | NM_011842.3:2579 | 6.310794527 | 6.615445725 | 0.304651198 |
| Cluster3 | Pgam1 | NM_023418.2 | Neuropathology | NM_023418.2:466 | 10.59920619 | 10.90556752 | 0.306361324 |
| Cluster3 | Lsr | NM_001164184.1 | Neuropathology | NM_001164184.1:445 | 9.40842528 | 9.716836606 | 0.308411325 |
| Cluster3 | Lclat1 | NM_001177967.1 | Neuropathology | NM_001177967.1:2646 | 10.18032567 | 10.50118534 | 0.320859667 |
| Cluster3 | Ube2n | NM_080560.3 | Neuropathology | NM_080560.3:2376 | 9.29872704 | 9.627305881 | 0.328578841 |
| Cluster3 | Camk2d | NM_001025439.1 | Neuropathology | NM_001025439.1:1315 | 11.58674364 | 11.91677892 | 0.33003528 |
| Cluster3 | Igf1r | NM_010513.2 | Neuropathology | NM_010513.2:3390 | 10.15564679 | 10.48636242 | 0.330715636 |
| Cluster3 | Inpp5f | NM_178641.5 | Neuropathology | NM_178641.5:2806 | 9.488583292 | 9.828176162 | 0.339592869 |
| Cluster3 | Cds1 | NM_173370.3 | Neuropathology | NM_173370.3:2232 | 10.90237888 | 11.25497839 | 0.35259951 |
| Cluster3 | Homer1 | NM_147176.2 | Neuropathology | NM_147176.2:1165 | 7.890841848 | 8.257552847 | 0.366711 |
| Cluster3 | Xbp1 | NM_013842.2 | Neuropathology | NM_013842.2:825 | 10.98864919 | 11.38529351 | 0.396644326 |
| Cluster3 | Gfpt1 | NM_013528.3 | Neuropathology | NM_013528.3:718 | 9.977537525 | 10.3963586 | 0.418821078 |
| Cluster3 | Taf9 | NM_027139.5 | Neuropathology | NM_027139.5:324 | 9.378273177 | 9.798050515 | 0.419777337 |
| Cluster3 | Ppp3ca | NM_008913.4 | Neuropathology | NM_008913.4:1675 | 10.3696028 | 10.81038285 | 0.440780052 |
| Cluster3 | Car2 | NM_009801.4 | Neuropathology | NM_009801.4:437 | 10.00330708 | 10.48936129 | 0.486054216 |
| Cluster3 | Epha7 | NM_001122889.1 | Neuropathology | NM_001122889.1:844 | 9.102946767 | 9.87475855 | 0.771811783 |

**Supplementary Table 5. Real-time PCR primer information for ChIP-seq validation.**

- Primer information for *Bdnf* genes (Mouse)

| Primer information for ChIP-qPCR analysis | | |
|---|---|---|
| Name | Sequence | Amplicon locus (mm9) |
| -m3246 S | 5′-TGGCCAGAAACTGTTGACAAAGTC-3′ | chr2:109,512,735-109,512,808 |
| -m3246 AS | 5′-GGGAGGATGGTCTGTATGCGAA-3′ | |
| -m477 S | 5′-GAATCGGGTTTACCCACTGCAAG-3′ | chr2:109,515,618-109,515,756 |
| -m477 AS | 5′-ACGGTTGTCAGACAAGCATC-3′ | |
| +m383 S | 5′-AAAGGACGCGTAGTGGAGAGG-3′ | chr2:109,516,618-109,516,717 |
| +m383 AS | 5′-CCCAGGTTCTCACCTAGGTC-3′ | |
| +m1123 S | 5′-GGCCGGATGCTTCATTGAGC-3′ | chr2:109,517,358-109,517,447 |
| +m1123 AS | 5′-GAAAGGACCTTCCACTCCGG-3′ | |
| +m3353 S | 5′-TCAACCTGTGTAAGCCGCTGC-3′ | chr2:109,519,688-109,519,807 |
| +m3353 AS | 5′-GTTTCCCCTAGAGGACAGGC-3′ | |

- Primer information for *HOXB* cluster genes (Human)

| Primer information for ChIP-qPCR analysis | | |
|---|---|---|
| Name | Sequence | Amplicon locus (hg19) |
| HOXB3 Gene body S | 5′-GCATTTTCAGCGGTCTCTTC-3′ | chr17:46,662,566-46,662,717 |
| HOXB3 Gene body AS | 5′-AGAAATGCCCTTCTCCAGGT-3′ | |
| HOXB3 TSS S | 5′- CCAGAATTGCAAGGGGTAAA-3′ | chr17:46,667,444-46,667,598 |
| HOXB3 TSS AS | 5′- TTTAACTGCTCGCTGTGGTG -3′ | |
| HOXB5 TSS S | 5′-CTGAGGTCCATCCCATTGTAA-3′ | chr17:46,670,881-46,671,087 |
| HOXB5 TSS AS | 5′-TAACGACCACGATCCACAAA-3′ | |

**Supplementary Table 6. Real-time PCR primer information for transcription quantification.**

- Primer information for validation of gene expression changes (mouse)

| Primer information for qRT-PCR analysis | | |
|---|---|---|
| Name | Sequence | Amplicon locus (mm9) |
| Zfp62 S | 5′-ATCCAAGAAGGGCCTCCAAC-3′ | chr11:49,031,638-49,031,756 |
| Zfp62 AS | 5′-TGTGATCTTCCTACCACAAACCAT-3′ | |
| Phf20l1 S | 5′-GTACAGACTCAGTGTTCCCACA-3′ | chr15:66,475,675-66,475,745 |
| Phf20l1 AS | 5′-TTTCCACTAGGGGAGTCCGT-3′ | |
| Rxra S | 5′-ATCTGTGTTGCTACCTGCCC-3′ | chr2:27,618,657-27,618,753 |
| Rxra AS | 5′-CTGAGTCCCTCCGTGTGAAC-3′ | |
| Jph3 S | 5′-GAGCATGTGCCGTTCGTTTT-3′ | chr8:124,314,164-124,314,263 |
| Jph3 AS | 5′-TCAGTGAGAGGTGACGTCCT-3′ | |
| Srm S | 5′-GTGGTCCAGTGCGAGATTGA-3′ | chr4:147,966,660-147,967,475 |
| Srm AS | 5′-CCACGTGGAGAGTCAGCTTT-3′ | |
| Chga S | 5′-TCAGGCCTTTCCAGAGCCTA-3′ | chr12:103,799,980-103,800,077 |
| Chga AS | 5′-GGCTGGTTGGTGATTGGGTA-3′ | |
| Grhpr S | 5′-CTGACCCTCAAGAACTGCGT-3′ | chr4:45,001,904-45,003,302 |
| Grhpr AS | 5′-AGGACATGGTGTTGCGAGTT-3′ | |
| Gapdh S | 5'-CATCACTGCCACCCAGAAGACTG-3' | chr1:182,257,618-182,257,770 |
| Gapdh AS | 5'-ATGCCAGTGAGCTTCCCGTTCAG-3' | |

- Primer information for analysis of gene expression change (Human)

| Primer information for qRT-PCR analysis | | |
|---|---|---|
| Name | Sequence | Amplicon locus (hg19) |
| MECP2 S | 5'-ACTTCTGGCCCTGGTTAGGT-3' | chrX:153,295,583-153,295,840 |
| MECP2 AS | 5'-CCGTGACCGAGAGAGTTAGC-3' | |
| GAPDH S | 5'-GATCATCAGCAATGCCTCCT-3' | chr12:6,646,374-6,646,755 |
| GAPDH AS | 5'-TGTGGTCATGAGTCCTTCCA-3' | |

**Supplementary Table 7. Primer information for cloning.**

| Name | Sequence |
|---|---|
| MeCP2 Full XhoI For | 5′-AAAA CTCGAG GTAGCTGGGATGTTAGGGCTCA-3′ |
| MeCP2 Full NotI Rev | 5′-AAAA GCGGCCGC TCA GCTAACTCTCTCGGTCACG-3′ |
| MeCP2 HMGD1/MBD NotI Rev. | 5′-AAAA GCGGCCGC TCA GCTCCCTCTCCCAGTTACCG-3′ |
| MeCP2 CTD XhoI For | 5′-AAAA CTCGAG ACGGTCAGCATCGAGGTCAA-3′ |
| MeCP2 MBD/HMGD2 XhoI For | 5′-AAAA CTCGAG AGCCCCTCCCGGCGAGA-3′ |
| MeCP2 MBD/HMGD2 NotI Rev | 5′-AAAA GCGGCCGC TCA CTCCCGGGTCTTGCGCTT-3′ |
| MeCP2 TRD/CTD XhoI For | 5′-AAAA CTCGAG CAGGTGAAAAGGGTCCTGGAG-3′ |
| MeCP2 Full EcoRI For | 5′-AAAA GAATTC GTAGCTGGGATGTTAGGGCTCA-3′ |
| MeCP2 MBD EcoRI For | 5′-AAAA GAATTC GAAGCTTCTGCCTCCCCCA-3′ |

## Supplementary Methods

***Peak centered aggregation plot of MeCP2 ChIP-seq and MNase-seq (Figs. 1D and E).***

Using PING[5,6], either nucleosome occupancy or MeCP2 binding loci were defined. For the MNase-seq analysis, "MNase" as the default of the datatype in the postPING() option (alpha2=98; beta2=200000) were used. For MeCP2 ChIP-seq analysis, the combined MeCP2 ChIP-seq data from the two biological replicates were analyzed with "sonicated" in the postPING() option (alpha2=100; beta2=100000). Then Seqplots[7] were used to plot average signal intensity of MeCP2, histone H1, and nucleosome across the summits of either MeCP2 ChIP enrichment or MNase-seq. Mean signal densities were calculated for 10 bp windows and visualized with Z-score transformation across the summits extended by 1 kb on either side.

***Correlation analysis among Mnase-seq, MeCP2 ChIP-seq, histone H1 ChIP-seq, Input library generated (Fig. 1F).***

The correlation between Mnase-seq, MeCP2 ChIP-seq, histone H1 ChIP-seq and input library was analyzed by multiBamSummary with –bs 150 and –n 1000, and plotted with plotCorrelation of Deeptools[8] package.

***Classification MeCP2 binding and nucleosome positioning (Fig. 2B).***

Overlapping or non-overlapping regions between the nucleosome occupied loci and MeCP2 attachment region, determined by the program PING[5,6], were analyzed using the bedtools[9] intersect (v2.24.0) command. Loci with over 75% overlap between nucleosome occupied region and MeCP2-enriched region were considered to be MeCP2-enriched mononucleosome loci, while loci with less than 75% overlap were considered to be either MeCP2-absent mononucleosomes loci or nucleosome-free MeCP2 binding loci.

***DNA Methylation level comparison on nucleosome only, nucleosome overlap with MeCP2, and nucleosome free MeCP2 region (Fig. 2C).***

To determine correlation between DNA methylation with previously determined clusters, after binning the genome to the randomly chosen 10,000 loci from each nucleosome-only, nucleosome overlap with MeCP2, and nucleosome free MeCP2 binding loci, the methylation level of each CpG, CAG, CAH in the genome was estimated as the ratio $I(x) = n_m(x) / n_{tot}(x)$ where $n_m(x)$ is the number of reads supporting methylation at position x and $n_{tot}(x)$ is the total number of covering reads. We compared the average methylation level I(i) of each bin i with Kruskal-Wallis test, followed by two-tailed Mann-Whitney test with Bonferroni correction using SPSS24.

***Differential histone PTMs analysis on nucleosome only, nucleosome overlap with MeCP2, and nucleosome free MeCP2 regions (Figs. 2D to F).***

To analyze histone PTMs, MeCP2-absent mononucleosome loci, MeCP2-enriched ≘ mononucleosome loci, and nucleosome-free MeCP2 binding loci on Chr19, signal intensity of MNase-seq, Input, MeCP2, H3K27me3, and H3K9ac ChIP-seq were ordered by MeCP2 ChIP/Input signal using Easeq[10] (Fig. 2D). The mean of input normalized histone PTMs ChIP-seq in nucleosome only, nucleosome overlap with MeCP2, and nucleosome free MeCP2 loci on chr19 were compared using Kruskal-Wallis test, followed by two-tailed Mann-Whitney test with Bonferroni correction using SPSS24 (Figs. 2E and F). $p = 0.00$ were reported as $p < 0.0001$.

***Correlation analysis among H3K27me3, H3K9ac, MeCP2 ChIP-seq, and Input library generated from mouse OE tissue (Fig. 2G).***

The correlations between the H3K27me3, H3K9ac, and MeCP2 ChIP-seq libraries were analyzed by multiBamSummary with –bs 1000 and –n 1000, and correlation plot was generated with plotCorrelation of Deeptools[8] package.

***Comparison of H3K27me3 enrichment by ChIP-seq between DMSO and GSK343 treated SH-SY5Y cells (Fig. 4C).***

Reads counts of H3K27me3 ChIPed DNA from DMSO- or GSK343-treated SH-SY5Y cells were evaluated within the loci used for targeted bisulfite sequencing with RRPM normalization. Differences of H3K27me3 signals between the DMSO- and GSK343-treated samples are shown in a scatter plot. The tested loci were arbitrarily categorized according to the levels of H3K27me3 reduction in GSK343 treatment: unchanged (n = 333,212), moderate reduction (n = 16,434), severe reduction (n = 691). Since H3K27me3 shows unidirectional signal change in GSK343 treated samples compared to DMSO controls (Supplementary Fig. 7A), RRPM normalization, against drosophila S2 cells, was applied to H3K27me3 read count normalization for further comparative analysis.

***Analysis of MeCP2 binding change in correlation with the reduction of H3K27me3 (Fig. 4D).***

Reads counts of MeCP2 ChIP-seq within the regions from previously defined class in Fig. 4C were normalized to RPM. The adjusted reads count from the DMSO- or GSK343-treated sample were separately evaluated, and then the differences were statistically evaluated by Wilcoxon signed-rank test using SPSS24. True $p = 0.00$ were reported as $p < 0.0001$. Since MeCP2 shows bidirectional signal change (Supplementary Fig. 7B), RPM normalization was applied to MeCP2 analysis.

***MeCP2 binding difference between HCT116 and DKO1 at DMRs (Fig. 5A).***

Using metilene[11], differentially methylated regions (DMRs) were identified from WGBS-seq data (GSM1465024 and GSM1465025)[12]. MeCP2 ChIP-seq read counts within DMRs were normalized and presented as RPM. The normalized read counts of the HCT116 or DKO1 were statistically evaluated by Wilcoxon signed-rank test using SPSS24. True $p = 0.00$ were reported as $p < 0.0001$. Since MeCP2 shows bidirectional signal change (Supplementary Fig. 8B), RPM normalization was applied to MeCP2 analysis.

***Correlation analysis among H3K27me3, MeCP2 ChIP-seq, and Input library generated from HCT116 and DKO1 cells (Fig. 5B).***

The correlations between H3K27me3 and MeCP2 ChIP-seq libraries were analyzed by multiBamSummary with –bs 200 and –n 10000, and correlation plot was generated with plotCorrelation of Deeptools package.

***Differential H3K27me3 peaks analysis between HCT116 and DKO1 cells (Fig. 5C).***

Using MACS2[13] with "macs2 callpeak" in the default with –q 0.01 –extsize 150, H3K27me3 modification loci of HCT116 and DKO1 were defined. H3K27me3 modification difference between HCT116 and DKO1 was analyzed with "macs2 bdgdiff" with –g 60 –l 120.

***Meta-gene analysis of H3K27me3 and MeCP2 ChIP-seq for differential H3K27me3 modification loci between HCT116 and DKO1 (Figs. 5D and E).***

Input normalized H3K27me3 and MeCP2 bigwig files were generated using bamCompare in deepTools package with the options –normalizeUsing CPM –extendReads 200. Using Seqplots[7], average signal intensities of H3K27me3 modification and MeCP2 binding were plotted for differential H3K27me3 modification loci between HCT116 and DKO1, defined by MACS2[13]. Mean signal densities were calculated for 10 bp windows and visualized across the summits extended by 1 kb on either side. Since both H3K27me3 and MeCP2 show bidirectional signal change (Fig. 5C and Supplementary Fig. 8B), RPM normalization was applied to both H3K27me3 and MeCP2 analysis.

***Representative examples of differential H3K27me3 modification and MeCP2 binding loci in DMR (Fig. 5F).***

For DNA methylation information, Methylation % and DMR loci were generated from WGBS-seq data (GSM1465024 and GSM1465025)[12]. For H3K27me3 modification and MeCP2 binding information, input normalized H3K27me3 and MeCP2 bigwig files were generated using bamCompare of deepTools package with the options –normalizeUsing SES –extendReads 200.

***Analysis of the association between MeCP2 binding (Fig. 6A) and annotated gene structure regions (Fig. 6B).***

Using annotatepeaks.pl function from HOMER, the MeCP2 binding peaks were annotated to the nearest mm9 refseq gene (Fig. 6A). For the associations analysis between MeCP2 peaks and genomic regions, we used the permTest function, within regioneR package[14], with ntimes = 1000, randomize.function = circularRandomizedRegions, evaluate.function = numOverlaps, and mc.set.seed = FALSE. Also, local Z-scores were calculated with window = 2000, step = 50, count.once = TRUE (Fig. 6B).

***Co-localization analysis of MeCP2, H3K27me3, and H3K9ac peaks (Fig. 6D).***

Using PING[5,6] with "sonicated" in the postPING() option (alpha2=100; beta2=100000), H3K27me3 and H3K9ac modification loci were defined. Co-localization between the histone PTMs and MeCP2 were analyzed using the bedtools[9] intersect (v2.24.0) command.

***Meta-gene analysis of MeCP2, H3K27me3, H3K9ac ChIP-seq and bisulfite sequencing around TSS and Enhancer regions (Figs. 6E, F, G and H).***

Seqplots[7] was used to plot average signal intensity of MeCP2 binding, H3K27me3 and H3K9ac modification and DNA methylation level from bisulfite sequencing. For the meta-gene analysis around TSS, mean signal densities were calculated for 10 bp windows and visualized with Z-score transformation across the summits extended by 1 kb up- and 4kb downstream of the TSS. Regarding the meta-gene analysis around enhancer, first, we defined enhancer regions. From the published DNase-seq (GSM1267295), H3K4me1 (GSM1267287 and 1267288), and H3K27ac (GSM1267289 and 1267290) ChIP-seq reads[4], referenced by previous studies [15,16], loci of DHS+ / H3K4me1+ / H3K27ac+ defined as an OE specific enhancer. Next, mean signal densities were calculated for 10 bp windows and visualized with Z-score transformation across the summits extended by 3 kb on either side of the enhancer.

***Co-local clustering of MeCP2 and histone PTM (Fig. 7A) and gene expression analysis of the clusters (Fig. 7B).***

SuperSom clustering, within Seqplot[7] Package, were used for clustering of the MeCP2, H3K27me3, and H3K9ac enrichment signal across the upstream of 1 kb and downstream of 5 kb for each gene. The clustering was visualized

with decreasing order of heatmap (Fig. 7A). The log10 scaled RPKM values from three replicate of WT RNA-seq for the clustered genes were plotted and compared using Kruskal-Wallis test, followed by two-tailed Mann-Whitney test with Bonferroni correction using SPSS24 (Fig. 7B).

***Gene set enrichment analysis of differentially expressing genes of WT and Mecp2 KO (Figs. 7C to E).***

For top 100 genes from each group, gene set enrichment analysis (GSEA) was performed as described[17]. We excluded genes not detected in RNA-seq analyses and used following parameters. Number of permutations: 1000, Collapse dataset to gene symbols: False, Enrichment statistic: Classic, Max gene set size: 5000, Min gene set size: 15, Collapsing mode for probe sets>1 gene: Max_probe, Normalisation mode: meandiv, Omit features with no symbol: True. $p = 0.00$ were reported as $p < 0.001$.

***Amplification free differential gene expression analysis between WT and Mecp2 KO (Figs. 7F to H).***

The transcription levels of the biological two replicates from WT and *Mecp2* KO were measured in nCounter Mouse Neuropathogy Panel Kit. Gene expression values in nCounter Mouse Neuropathology Panel Kit were normalized with positive ERCC spike-in and reference gene, Abl1. Among the differentially expressed genes between WT and *Mecp2* KO, genes expression value outside the 95% confidence interval were considered to be either an increase or a decrease in gene expression.

***Differential expression analysis (Figs. 8A to C).***

FDR < 0.05 with fold change > 1.3 or < -1.3 were used to determine genes that were up- or down-regulated genes. For the significantly changed genes between WT and *Mecp2* KO, input normalized enrichment of MeCP2, H3K27me3, or H3K9ac were compared in for TSS flanking regions (−1 kb to +1 kb), gene bodies except regulatory regions (+1 kb up to +10 kb), and flanking region of the genes (−6 kb to -1 kb, and TES to +5 kb) by Mann-Whitney test using SPSS24.

***Correlation analysis of targeted bisulfite sequencing between DMSO- and GSK343- treated SH-SY5Y cells (Supplementary Fig. 6H).***

The correlation between DNA methylation of DMSO- and GSK343-treated SH-SY5Y cells was analyzed and visualized by Methylkit[18] R package with reduced-representation bisulfite sequencing (RRBS) module.

## Supplementary References


1. Young, M.D. *et al.* ChIP-seq analysis reveals distinct H3K27me3 profiles that correlate with transcriptional activity. *Nucleic Acids Res* **39**, 7415-7427 (2011).
2. Millán-Ariño, L. *et al.* Mapping of six somatic linker histone H1 variants in human breast cancer cells uncovers specific features of H1. 2. *Nucleic Acids Res* **42**, 4474-4493 (2014).
3. Karmodiya, K., Krebs, A.R., Oulad-Abdelghani, M., Kimura, H. & Tora, L. H3K9 and H3K14 acetylation co-occur at many gene regulatory elements, while H3K14ac marks a subset of inactive inducible promoters in mouse embryonic stem cells. *BMC Genomics* **13**, 424 (2012).
4. Markenscoff-Papadimitriou, E. *et al.* Enhancer interaction networks as a means for singular olfactory receptor expression. *Cell* **159**, 543-57 (2014).
5. Woo, S., Zhang, X., Sauteraud, R., Robert, F. & Gottardo, R. PING 2.0: an R/Bioconductor package for nucleosome positioning using next-generation sequencing data. *Bioinformatics* **29**, 2049-2050 (2013).
6. Zhang, X., Robertson, G., Woo, S., Hoffman, B.G. & Gottardo, R. Probabilistic inference for nucleosome positioning with MNase-based or sonicated short-read data. *PLoS One* **7**, e32095 (2012).
7. Stempor, P. & Ahringer, J. SeqPlots-Interactive software for exploratory data analyses, pattern discovery and visualization in genomics. *Wellcome open research* **1**(2016).
8. Ramirez, F. *et al.* deepTools2: a next generation web server for deep-sequencing data analysis. *Nucleic Acids Res* **44**, W160-165 (2016).
9. Quinlan, A.R. & Hall, I.M. BEDTools: a flexible suite of utilities for comparing genomic features. *Bioinformatics* **26**, 841-2 (2010).
10. Lerdrup, M., Johansen, J.V., Agrawal-Singh, S. & Hansen, K. An interactive environment for agile analysis and visualization of ChIP-sequencing data. *Nat Struct Mol Biol* **23**, 349-357 (2016).
11. Juhling, F. *et al.* metilene: fast and sensitive calling of differentially methylated regions from bisulfite sequencing data. *Genome Res* **26**, 256-62 (2016).
12. Blattler, A. *et al.* Global loss of DNA methylation uncovers intronic enhancers in genes showing expression changes. *Genome Biol* **15**, 469 (2014).
13. Zhang, Y. *et al.* Model-based analysis of ChIP-Seq (MACS). *Genome Biol* **9**, R137 (2008).
14. Gel, B. *et al.* regioneR: an R/Bioconductor package for the association analysis of genomic regions based on permutation tests. *Bioinformatics* **32**, 289-291 (2016).
15. Heintzman, N.D. *et al.* Histone modifications at human enhancers reflect global cell-type-specific gene expression. *Nature* **459**, 108-12 (2009).
16. Zentner, G.E., Tesar, P.J. & Scacheri, P.C. Epigenetic signatures distinguish multiple classes of enhancers with distinct cellular functions. *Genome Res* **21**, 1273-83 (2011).
17. Subramanian, A. *et al.* Gene set enrichment analysis: a knowledge-based approach for interpreting genome-wide expression profiles. *Proc Natl Acad Sci U S A* **102**, 15545-15550 (2005).
18. Akalin, A. *et al.* methylKit: a comprehensive R package for the analysis of genome-wide DNA methylation profiles. *Genome Biol* **13**, R87 (2012).
