# Supplementary material for: MeCP2 regulates gene expression through recognition of H3K27me3
Source: Nat Commun. 2020 Jun 19;11:3140. doi: 10.1038/s41467-020-16907-0 (PMC7305159; doi:10.1038/s41467-020-16907-0)
Supplement: Supplementary file 3 — Reporting Summary [file 41467_2020_16907_MOESM3_ESM.pdf]

nature research

Corresponding author(s): Takbum Ohn & Qizhi Gong
Last updated by author(s): May 9, 2020

Reporting Summary

Nature Research wishes to improve the reproducibility of the work that we publish. This form provides structure for consistency and transparency in reporting. For further information on Nature Research policies, see Authors & Referees and the Editorial Policy Checklist.

Statistics

For all statistical analyses, confirm that the following items are present in the figure legend, table legend, main text, or Methods section.

n/a	Confirmed	
☐	☒	The exact sample size (n) for each experimental group/condition, given as a discrete number and unit of measurement
☐	☒	A statement on whether measurements were taken from distinct samples or whether the same sample was measured repeatedly
☐	☒	The statistical test(s) used AND whether they are one- or two-sided<br>*Only common tests should be described solely by name; describe more complex techniques in the Methods section.*
☐	☒	A description of all covariates tested
☐	☒	A description of any assumptions or corrections, such as tests of normality and adjustment for multiple comparisons
☐	☒	A full description of the statistical parameters including central tendency (e.g. means) or other basic estimates (e.g. regression coefficient) AND variation (e.g. standard deviation) or associated estimates of uncertainty (e.g. confidence intervals)
☐	☒	For null hypothesis testing, the test statistic (e.g. F, t, r) with confidence intervals, effect sizes, degrees of freedom and P value noted<br>*Give P values as exact values whenever suitable.*
☒	☐	For Bayesian analysis, information on the choice of priors and Markov chain Monte Carlo settings
☒	☐	For hierarchical and complex designs, identification of the appropriate level for tests and full reporting of outcomes
☒	☐	Estimates of effect sizes (e.g. Cohen's d, Pearson's r), indicating how they were calculated

Our web collection on statistics for biologists contains articles on many of the points above.

Software and code

Policy information about availability of computer code

Data collection	The built-in softwares provided with instrument was used for data collection: Bio-Rad CFX96 Real-time PCR detection system, Pyrosequencing PSQ96 HS System, NIKON NIS-Elements Advanced Research, Illumina HiSeq 2000, HiSeq 2500 and NextSeq500.
Data analysis	Bowtie 2 ver.2.4 (for Sequence alignment), SAMtools ver.1.1 (for PCR duplicates removal), BSseeker 2 ver.2.1 (for bisulfite sequencing alignment), TopHat 2 ver. 2.1 (for RNA-seq alignment), cufflinks ver. 2.2 and cuffdiff ver. 2.0.2 (for differential gene expression analysis), bedtools ver. 2.28.0 (for co-occupancy region analysis between nucleosome and MeCP2-enriched regions), deepTools ver. 2.0 (for conversion to bigWig file), R-package PING ver. 2.32.0 (for peak calling), HOMER ver. 4.11 (for peak annotation), R-package regioneR ver. 3.0 (for the associations analysis between MeCP2 peaks and genomic regions), SeqPlots ver 1.26 (for visualization of average signal intensity of ChIP-seq), Easeq ver 1.1 (for ChIP-seq data visualization), GSEA ver. 3.0 (for analysis of differential gene expression), nSolver Analysis Software 4.0 (for NanoString gene expression analysis), SPSS24 (for statistical analysis), GraphPad Prism5 (for figure generation), imagej (for Western blot quantification), Adobe Photoshop CS5 and 7.0.1 (for Western blot contrast adjustment), Qiagen QCpG (for Pyrosequencing data analysis)

For manuscripts utilizing custom algorithms or software that are central to the research but not yet described in published literature, software must be made available to editors/reviewers. We strongly encourage code deposition in a community repository (e.g. GitHub). See the Nature Research guidelines for submitting code & software for further information.

Data

Policy information about availability of data

All manuscripts must include a data availability statement. This statement should provide the following information, where applicable:

- Accession codes, unique identifiers, or web links for publicly available datasets
- A list of figures that have associated raw data
- A description of any restrictions on data availability

The data that support the findings of this study have been deposited to NCBI Gene Expression Omnibus under accession code GSE71126 and GSE122366.

Field-specific reporting

Please select the one below that is the best fit for your research. If you are not sure, read the appropriate sections before making your selection.

☒ Life sciences ☐ Behavioural & social sciences ☐ Ecological, evolutionary & environmental sciences

For a reference copy of the document with all sections, see nature.com/documents/nr-reporting-summary-flat.pdf

Life sciences study design

All studies must disclose on these points even when the disclosure is negative.

Sample size

The sample size was not pre-determined using statistical methods.

For ChIP-seq, all experiments are done with biological duplicates. Sample sizes were selected based on the references below.
Landt, S.G. et al. ChIP-seq guidelines and practices of the ENCODE and modENCODE consortia. Genome Res 22, 1813-31 (2012).

For RNA-seq, all experiments are done with biological triplicates. Sample sizes were selected based on the references below.
Schurch, N.J. et al., How many biological replicates are needed in an RNA-seq experiment and which differential expression tool should you use? RNA, 22, 839-51 (2016).

In all ChIP-qPCR and qRT-PCR experiments, each experiment was repeated at least once using a biologically independent experiment. Since these experiments are only used for verification of ChIP-seq or RNA-seq, the number of experiments is sufficient.

Data exclusions

We did not exclude any data points arbitrarily.

Replication

For ChIP-seq, each experiment was performed with two biological replicates. Reproducibility of ChIP-seq signals between two biological replicates was described in supplementary Fig. 2. The biological duplicates of ChIP-seq data showed a good agreement between the samples, at least r value greater than 0.87. ChIP-seq data was verified with ChIP-qPCR at the selected loci.

For RNA-seq, each experiment was performed with three biological replicate per genotype. The conclusion derived from the RNA-seq were validated with two bioloigical replicates using NanoString and RT-qPCR in selected genes.

For pull-down, Western blot, ChIP-qPCR and qRT-PCR experiments, each experiment was repeated at least once using biologically independent experiment. All reported data was successfully repeated.

Randomization

For experiments using mice, all control mice are of the same genetic background and are randomly chosen. When cell lines are used as biological samples, for treatment of pharmacological reagents, cell culture plates were randomly selected and processed in parallel. Bioinformatic analyses and comparisons are done with data obtained. When comparing data, biological triplicates are included to control covariates.

Blinding

When different biological samples are involved for phenotypic, biochemical, and bioinformatic comparisons, data collections are not possible to be blinded. When subset of data are chosen during bioinformatics analyses, the selection of data was random and determined by unbiased methods.

Reporting for specific materials, systems and methods

We require information from authors about some types of materials, experimental systems and methods used in many studies. Here, indicate whether each material, system or method listed is relevant to your study. If you are not sure if a list item applies to your research, read the appropriate section before selecting a response.

Materials & experimental systems

n/a	Involved in the study	
☐	☒	Antibodies
☐	☒	Eukaryotic cell lines
☒	☐	Palaeontology
☐	☒	Animals and other organisms
☒	☐	Human research participants
☒	☐	Clinical data

Methods

n/a	Involved in the study	
☐	☒	ChIP-seq
☒	☐	Flow cytometry
☒	☐	MRI-based neuroimaging

Antibodies

Antibodies used

Following antibodies were used for Western blotting:
rabbit anti-MeCP2 (Cell signaling, D4F3), 1:1000; Chicken anti-MeCP2 (custom antibody, gift from Dr J.M. LaSalle), 1:1000; goat anti-histone H1 (Santa cruz, sc-34464), 1:1000; rabbit anti-histone H3 (Cell signaling, D1H2), 1:3000; mouse anti-histone H4

(abcam , ab31830), 1:500; VeriBlot for IP secondary antibody (abcam, ab131366), 1:4000; Rabbit anti-Chicken HRP (IgY H&L) (abcam, ab6753), 1:50,000; rabbit anti-Ezh2 (Cell signaling, D2C9), 1:2000; rabbit anti-H3K27me3 (Cell signaling, C36B11), 1:2000; rabbit anti-GAPDH (Cell signaling, D16H11), 1:3000; mouse anti-GST (Santa Cruz, sc-138), 1:200; rabbit anti-DNMT1 (Cell signaling, D63A6) 1:2000; rabbit anti-DNMT3B (Cell signaling, E8A8A) 1:1000; mouse anti-actin (Santa cruz, sc-47778) 1:1000.

Following antibodies were used for immunofluorescent staining:
rabbit anti-H3K27me3 (Cell signaling, C36B11), 1:1000; mouse anti-MeCP2 (Sigma-Aldrich, Men-8), 1:1000.

Following antibodies were used for ChIP-qPCR / re-ChIP analysis:
(For olfactory neuroepithelia) 2μg of rabbit anti-MeCP2 (Diagenode, pAb-052-050); 2μg of goat anti-histone H1 (Santa Cruz, sc-34464); 2μg of rabbit IgG (Millipore, 12-370) as negative control.

Following antibodies were used for ChIP-seq analysis:
(For olfactory neuroepithelia) 50μg of rabbit anti-MeCP2 (Diagenode, pAb-052-050); 15μg of goat anti-histone H1 (Santa Cruz, sc-34464); 10μg of rabbit anti-H3K27me3 (Diagenode, pAb-069-050); 10μg of rabbit anti-H3K9ac (Diagenode, pAb-177-050).
(For SH-SY5Y, HCT116 and DKO1) 20μg of rabbit anti-MeCP2 (Diagenode, pAb-052-050); 10μg of rabbit anti-H3K27me3 (Diagenode, pAb-069-050).

Following antibodies were used for Co-IP assay:
(For olfactory epithelium) 4.8μg of rabbit anti-MeCP2 (Diagenode, pAb-052-050); 8μg of goat anti-histone H1 (Santa Cruz, sc-34464); 4.8μg of rabbit IgG (Millipore, 12-370) as negative control.
(For SH-SY5Y) 5μg of rabbit anti-MeCP2 (Diagenode, pAb-052-050); 5μg of rabbit anti-histone H3 (Abcam, ab1791); 5μg of rabbit IgG (Diagenode, kch-504-250) as negative control.

Following antibodies were used for Pulldown assay:
3μg of mouse anti-Flag (Sigma, F1804).

Validation

All antibodies except chicken anti-MeCP2 are commercially available antibodies. Validation for chicken anti-MeCP2 was fully described in the previous studies (Yasui et al., 2007).

Validation data for the ChIP grade of the MeCP2, H3K27me3, and H3K9ac antibodies are available
at https://www.diagenode.com/en/p/mecp2-polyclonal-antibody-classic-50-ug-42-ul. (ChIP-qPCR and Western blot analysis using whole cell extracts (40μg) from HeLa cells transfected with MeCP2 siRNA)

at https://www.diagenode.com/en/p/h3k27me3-polyclonal-antibody-classic-50-mg-34-ml. (ChIP-qPCR and cross reactivity test using dot blot analysis)

at https://www.diagenode.com/en/p/h3k9ac-polyclonal-antibody-classic-50-mg-47-ml. (ChIP-qPCR and cross reactivity test using dot blot analysis)

Validation for ChIP-seq grade of the MeCP2 antibody was fully described in the previous study (Rube et al, 2016). Validation for ChIP-seq grade of the histone H1, H3K9ac, and H3K27me3 antibody were analyzed by genomic characterization of ChIP-seq signal around TSS (supplementary Fig. 4). histone H1 binding and these histone modification patterns are consistent with published studies (Young et al., 2011; Millán-Ariño et al., 2014, and Karmodiya et al., 2012).

Eukaryotic cell lines

Policy information about cell lines

Cell line source(s)	SH-SY5Y (22266) from Korean Cell Line Bank, HCT116 cell line and DKO1 (HD R02-079) from Horizon, Drosophila S2 cells (CRL-1963) and 293T (CRL-3216) from ATCC.
Authentication	DKO1, DNMT1 (Δexons3-5/Δexons3-5); DNMT3B (-/-) of HCT116, was fully characterized in Supplementary Fig. 8A. Whole cell extracts were separated by SDS page and immune-blotted with anti-DNMT1 (Cell signaling, D63A6), and DNMT3b (Cell signaling, E8A8A). Western blot shows absence of DNMT1 and DNMT3B in DKO1.
Mycoplasma contamination	Cells were tested negative for mycoplasma contamination using mycoplasma detection kit (SouthernBiotech, 13100-01)
Commonly misidentified lines (See ICLAC register)	SH-SY5Y, 293T, HCT116, Drosophila S2 cell line are not listed in the commonly misidentified lines.

Animals and other organisms

Policy information about studies involving animals; ARRIVE guidelines recommended for reporting animal research

Laboratory animals

Mouse are housed in a 12 light / 12 dark cycle at 65-75°F (~18-23°C) with a humidity of 40-60%.

For ChIP-seq and ChIP-qPCR, 8-week-old male mice (C57BL/6J) were used to obtain main olfactory epithelium (MOE).

For RNA-seq and RT-qPCR, Male Mecp2-/y (KO) and Mecp2+/y (WT) littermates were obtained by crossing heterozygous Mecp2-/+ female (Jackson Laboratory strain: B6.129P2(C)-Mecp2tm1.1Bird/J, stock number 003890) with inbred C57BL/6J male. 8-week-old male Mecp2-/y (KO) and Mecp2+/y (WT) littermates were used to obtain main olfactory epithelium (MOE).

Wild animals	This study did not involve wild animals.
Field-collected samples	This study did not involve samples collected from the field.
Ethics oversight	Animal care and experimental procedures were approved by the Institutional Animal Care and Use Committee at University of California, Davis (UCD), and were in compliance with the National Institute of Health (NIH) policy. All animal experiments were performed at UCD.

Note that full information on the approval of the study protocol must also be provided in the manuscript.

ChIP-seq

Data deposition

☒ Confirm that both raw and final processed data have been deposited in a public database such as GEO.

☒ Confirm that you have deposited or provided access to graph files (e.g. BED files) for the called peaks.

Data access links
May remain private before publication.

MeCP2 ChIP-seq and MNase-seq from mouse OE tissue has been deposited at Gene Expression Omnibus (GSE:71126):
https://www.ncbi.nlm.nih.gov/geo/query/acc.cgi?acc=GSE71126

histone H1, H3K27me3 and H3K9ac ChIP-seq from mouse OE tissue has been deposited at Gene Expression Omnibus (GSE:122363)
https://www.ncbi.nlm.nih.gov/geo/query/acc.cgi?acc=GSE122363

MeCP2 and H3K27me3 ChIP-Rx from SH-SY5Y cells has been deposited at Gene Expression Omnibus (GSE:122364)
https://www.ncbi.nlm.nih.gov/geo/query/acc.cgi?acc=GSE122364

MeCP2 and H3K27me3 ChIP-Rx from HCT116 and DKO1 cells has been deposited at Gene Expression Omnibus (GSE:136118)
https://www.ncbi.nlm.nih.gov/geo/query/acc.cgi?acc=GSE136118

Targeted Bisulfite sequencing data from SH-SY5Y cells has been deposited at Gene Expression Omnibus (GSE:136119)
https://www.ncbi.nlm.nih.gov/geo/query/acc.cgi?acc=GSE136119

Files in database submission

Mouse OE ChIP-seq data:
- Raw sequencing data for Input, MeCP2 ChIP-seq and MNase-seq (GSM1827604, MeCP2_ChIP_WT_rep1; GSM1827605, MeCP2_ChIP_WT_rep2; GSM1827606, Input_WT_rep1; GSM1827607, Input_WT_rep2; GSM1827608, MNase_WT)
- bigwig files for Input, MeCP2 ChIP-seq and MNase-seq (GSE125585_Input_OE_merge.bw, GSE125585_MNase_seq_OE.bw, GSE125585_MeCP2_ChIP_OE_merge.bw)
- Peak files for MeCP2 ChIP-seq and MNase-seq (GSE125585_MNase_Peaks_by_PING.bed.gz, GSE125585_MeCP2_Peaks_by_PING.bed.gz)
- Raw sequencing data for histone H1, H3K9ac, and H3K27me3 ChIP-seq (GSM3465057, Histone_H1_ChIP_OE_rep1; GSM3465058, Histone_H1_ChIP_OE_rep2; GSM3465059, H3K9ac_ChIP_OE_rep1; GSM3465060, H3K9ac_ChIP_OE_rep2; GSM3465061 H3K27me3_ChIP_OE_rep1; GSM3465062, H3K27me3_ChIP_OE_rep2)
- bigwig files for histone H1, H3K9ac, and H3K27me3 ChIP-seq (GSM3465057_HistoneH1_ChIP_OE_merge.bw, GSM3465059_H3K9ac_ChIP_OE_rep1.bw, GSM3465060_H3K9ac_ChIP_OE_rep2.bw, GSM3465061_H3K27me3_ChIP_OE_rep1.bw, GSM3465062_H3K27me3_ChIP_OE_rep2.bw)

Human SH-SY5Y ChIP-Rx data:
- Raw sequencing data for Input, H3K27me3, and MeCP2 ChIP-Rx (GSM3465063, Input_ChIPRx_DMSO_rep1; GSM3465064, Input_ChIPRx_DMSO_rep2; GSM3465065, Input_ChIPRx_GSK343_rep1; GSM3465066, Input_ChIPRx_GSK343_rep2; GSM3465067, H3K27me3_ChIPRx_DMSO_rep1; GSM3465068, H3K27me3_ChIPRx_DMSO_rep2; GSM3465069, H3K27me3_ChIPRx_GSK343_rep1; GSM3465070, H3K27me3_ChIPRx_GSK343_rep2; GSM3489380, MeCP2_ChIPRx_DMSO_rep1; GSM3489381, MeCP2_ChIPRx_DMSO_rep2; GSM3489382, MeCP2_ChIPRx_GSK343_rep1; GSM3489383, MeCP2_ChIPRx_GSK343_rep2)
- bigwig files for Input, H3K27me3, and MeCP2 ChIP-Rx (GSE122364_DMSO_H3K27me3.ext200_RRPM.bw, GSE122364_DMSO_Input.ext200_RPM.bw, GSE122364_DMSO_MeCP2.ext200_RPM.bw, GSE122364_GSK343_H3K27me3.ext200_RRPM.bw, GSE122364_GSK343_Input.ext200_RPM.bw, GSE122364_GSK343_MeCP2.ext200_RPM.bw, GSE122364_H3K27me3_difference_GSK343_RRPM_vs_DMSO_RRPM.bw, GSE122364_MeCP2_difference_GSK343_RPM_vs_DMSO_RPM.bw)

Human HCT116 and DKO1 ChIP-Rx data:
- Raw sequencing data for Input, H3K27me3, and MeCP2 ChIP-Rx (GSM4041342, Input_ChIPRx_HCT116_rep1; GSM4041343, Input_ChIPRx_HCT116_rep2; GSM4041344, Input_ChIPRx_DKO1_rep1; GSM4041345, Input_ChIPRx_DKO1_rep2; GSM4041346, H3K27me3_ChIPRx_HCT116_rep1; GSM4041347, H3K27me3_ChIPRx_HCT116_rep2; GSM4041348, H3K27me3_ChIPRx_DKO1_rep1; GSM4041349, H3K27me3_ChIPRx_DKO1_rep2; GSM4041350, MeCP2_ChIPRx_HCT116_rep1; GSM4041351, MeCP2_ChIPRx_HCT116_rep2; GSM4041352, MeCP2_ChIPRx_DKO1_rep1; GSM4041353, MeCP2_ChIPRx_DKO1_rep2)
- bigwig files for Input, H3K27me3, and MeCP2 ChIP-Rx (GSE136118_DKO1_H3K27me3_Input_norm_CPM.bigwig, GSE136118_DKO1_MeCP2_Input_norm_CPM.bigwig, GSE136118_HCT116_H3K27me3_Input_norm_CPM.bigwig, GSE136118_HCT116_MeCP2_Input_norm_CPM.bigwig)

Human SH-SY5Y targeted BS-seq data:
- Raw sequencing (GSM4041354, Targeted_Bisulfite_DMSO_rep1; GSM4041355, Targeted_Bisulfite_DMSO_rep2;

GSM4041356, Targeted_Bisulfite_GSK343_rep1; GSM4041357, Targeted_Bisulfite_GSK343_rep2)
- Methylation report (GSM4041354_DMSO_re1_bismark_bt2_pe.deduplicated.CX_report.txt.gz,
GSM4041355_DMSO_re2_bismark_bt2_pe.deduplicated.CX_report.txt.gz,
GSM4041356_GSK343_re1_bismark_bt2_pe.deduplicated.CX_report.txt.gz,
GSM4041357_GSK343_re2_bismark_bt2_pe.deduplicated.CX_report.txt.gz)

Genome browser session
(e.g. UCSC)

Mouse OE ChIP-seq data: https://genome.ucsc.edu/cgi-bin/hgTracks?db=mm9&lastVirtModeType=default&lastVirtModeExtraState=&virtModeType=default&virtMode=0&nonVirtPosition=&position=chr3%3A45111749-45280294&hgsid=750835055_VXW1Ufpn9tkCarFf9JlUsIwoYCvH

Human SH-SY5Y ChIP-Rx data: https://genome.ucsc.edu/cgi-bin/hgTracks?db=hg19&lastVirtModeType=default&lastVirtModeExtraState=&virtModeType=default&virtMode=0&nonVirtPosition=&position=chr17%3A45360071%2D47917970&hgsid=750836351_qTS6Bxaj7wuae4F06t5cXZ1Iamyg

Human HCT116 and DKO1 ChIP-Rx data: https://genome.ucsc.edu/cgi-bin/hgTracks?db=hg19&lastVirtModeType=default&lastVirtModeExtraState=&virtModeType=default&virtMode=0&nonVirtPosition=&position=chr17%3A46658679%2D46681043&hgsid=752852509_JnBophaNXrmcF3pZjmJRPDNAjGCl

Methodology

Replicates

For ChIP-seq from mouse OE tissue, and ChIP-Rx from SH-SY5Y and HCT116 cells, we performed ChIP-seq analysis from two biological replicates with corresponding inputs to each ChIP sample. Reproducibility of ChIP-seq signals between the two biological replicates was analyzed in supplementary Fig. 2.

Sequencing depth

Total number of reads and mappability for each sample is summarized in supplementary table1.

Antibodies

MeCP2 (Diagenode, pAb-052-050)
histone H1 (Santa Cruz, sc-34464)
H3K27me3 (Diagenode, pAb-069-050)
H3K9ac (Diagenode, pAb-177-050)

Peak calling parameters

Using PING, either nucleosome occupancy or MeCP2 binding loci were defined. For the MNase-seq analysis, “MNase” as the default of the datatype in the postPING() option (alpha2=98; beta2=200000) were used. For MeCP2 ChIP-seq analysis, the combined MeCP2 ChIP-seq data from the two biological replicates were analyzed with “sonicated” in the postPING() option (alpha2=100; beta2=100000).

Using MACS2 with “macs2 callpeak” in the default with –q 0.01 –extsize 150, H3K27me3 modification and MeCP2 binding loci of HCT116 and DKO1 were defined. H3K27me3 modification or MeCP2 binding difference between HCT116 and DKO1 was analyzed with “macs2 bdgdiff” with –g 60 –l 120. The same applies to ChIP-seq analyses in SH-SY5Y cell line.

Data quality

Summary of the total number of aligned and unique reads for all data were listed in supplementary table1.
Data quality of MeCP2 ChIP-seq and MNase-seq from mouse OE tissue were fully described in the previous study (Rube et al, 2016). Data quality of histone H1, H3K9ac, and H3K27me3 ChIP-seq from mouse OE tissue were analyzed by genomic characterization of ChIP-seq signal around TSS (supplementary Fig. 4). histone H1 binding and these histone modification patterns are consistent with published studies (Young et al., 2011; Millán-Ariño et al., 2014, and Karmodiya et al., 2012).

Software

Bowtie 2 (for Sequence alignment), SAMtools (for PCR duplicates removal), bedtools (for co-occupancy region analysis between nucleosome and MeCP2-enriched regions), deepTools (for conversion to bigWig file), R-package PING (for peak calling), HOMER (for peak annotation), R-package regioneR (for the associations analysis between MeCP2 peaks and genomic regions), MACS2 (for peak calling), SeqPlots (for visualization of average signal intensity of ChIP-seq), Bismark and Methylkit (for BS-sequencing analysis and visualization), Metilene (for differentially methylated regions analysis).
