# Supplementary material for: MeCP2 regulates gene expression through recognition of H3K27me3
Source: Nat Commun. 2020 Jun 19;11:3140. doi: 10.1038/s41467-020-16907-0 (PMC7305159; doi:10.1038/s41467-020-16907-0)

Source Data

MeCP2 regulates gene expression through recognition of H3K27me3

Lee *et al.*

(A) WB in Figure 1B

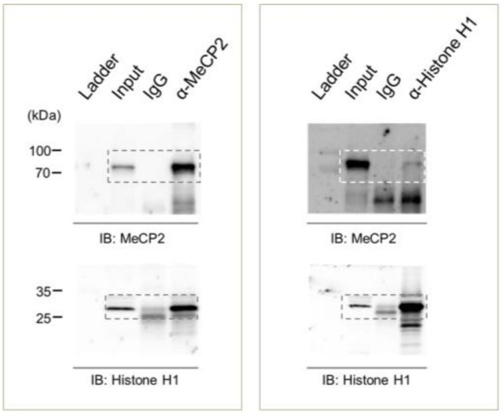

(B) WB in Figure 1C

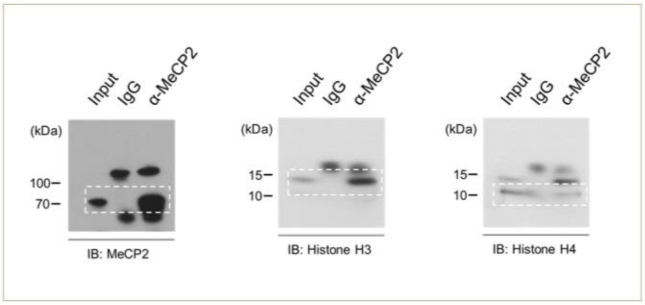

(C) WB in Figure 3A

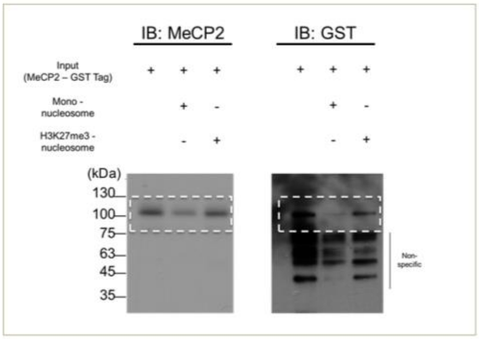

(D) WB in Figure 3C

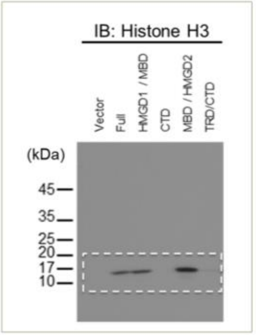

(E) WB in Figure 3D

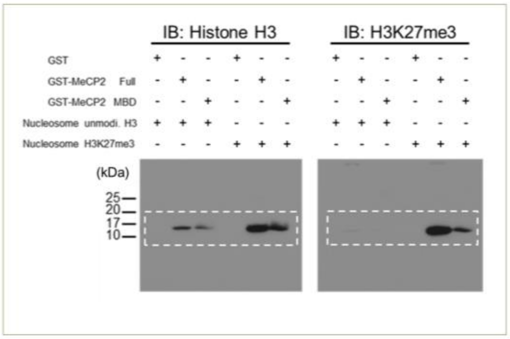

(F) WB in Figure 3E

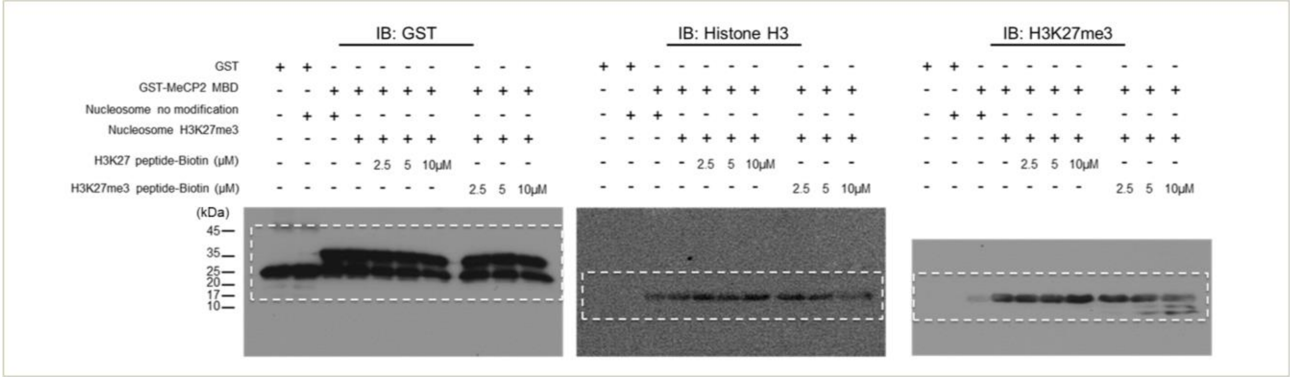

(G) WB in Figure 4A

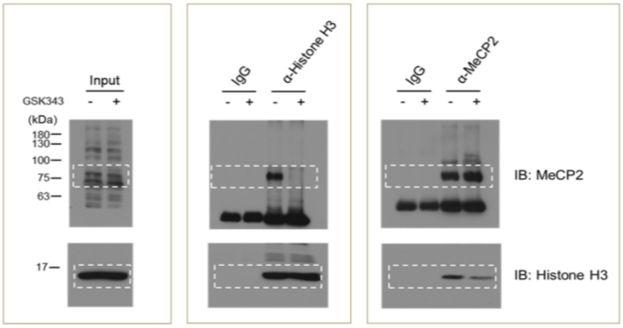

(H) Supplementary Fig.1A

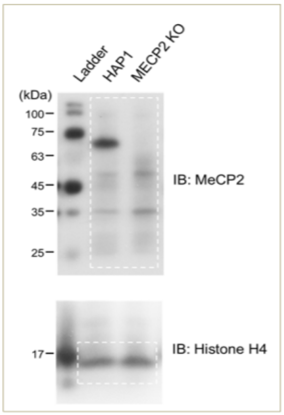

(I) Supplementary Fig. 6A

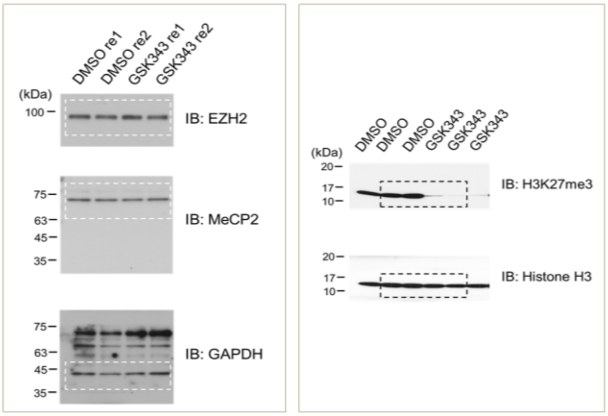

(J) Supplementary Fig. 8A

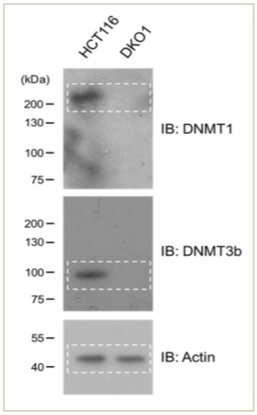

Supplement: Supplementary file 4 — Source Data [file 41467_2020_16907_MOESM4_ESM.pdf]
